# A Systematic Literature Review on the Burden of Disease for Patients With Moderate to Severe Acute Ischemic Stroke

**DOI:** 10.1097/MD.0000000000041249

**Published:** 2025-01-17

**Authors:** Terry Quinn, Kazuo Kitagawa, Thomas Leung, Carlos Molina, Alejandro Rabinstein, Roy Bentley, Owen Henry, Maria Heuser, Vedes Nair, Jeffrey Saver

**Affiliations:** a Reader and Honorary Consultant Physician in Stroke at the University of Glasgow, Glasgow, United Kingdom; b Professor and Chairman at Tokyo Women’s Medical University, Tokyo, Japan; c Professor of Neurology, Assistant Dean, Faculty of Medicine; Medical Director, Kwok Tak Seng Centre for Stroke Research and Intervention, The Chinese University of Hong Kong, Hong Kong; d Medical Director of the Stroke Unit and Brain Hemodynamics at Vall d’Hebron Hospital, Barcelona, Spain; e Medical Director and Professor of Neurology at the Mayo Clinic Stroke Center, MN; f Vice President, Global Scientific Operations, Shionogi Inc, NJ; g Value Analyst, Adelphi Values PROVE™, Bollington, United Kingdom; h Associate Value Consultant, Adelphi Values PROVE™, Bollington, United Kingdom; i Value Analyst, Adelphi Values PROVE™, Bollington, United Kingdom; j Professor and SA Vice-Chair of Neurology at the University of California, Los Angeles, CA

**Keywords:** acute ischemic stroke, burden, cost-effectiveness, impairment, quality-of-life

## Abstract

**Background:**

A vast amount of literature is available on the burden of acute ischemic stroke (AIS). Yet, most information on AIS burden does not stratify by stroke severity, and the inclusion of mild strokes (National Institute of Health Stroke Scale < 5) might obscure the true impact of moderate-to-severe AIS. Therefore, it is important to understand the literature as it pertains to the epidemiological, clinical, humanistic, and economic burden of moderate-to-severe AIS from a global perspective.

**Methods:**

A systematic literature review (SLR) was conducted, including articles published between January 2015 and June 2023. The clinical burden search focused on patients with moderate or severe AIS. Due to the paucity of evidence, the humanistic and economic burdens were evaluated based on overall AIS studies. Abstract and full-text screening were conducted by 2 reviewers, with data extraction completed by 1 reviewer. In all, 136 studies were included in the SLR.

**Results:**

AIS caused a substantial burden for patients and the healthcare system. The clinical burden of AIS (specifically severe AIS) resulted in high mortality and worse functional outcomes across multiple demographics (female sex, older age, and patients with comorbidities). The economic burden of overall AIS was substantial, with inpatient costs as the primary driver (a mean or median stay of 7 days). The highest inpatient costs were reported in South Korea ($45,180) and the United States ($38,470).

**Conclusions:**

The review highlighted the huge burden of moderate-to-severe AIS, with patients experiencing worse outcomes with increased stroke severity. Further focus is needed on outcomes relating to moderate-to-severe AIS to fully understand the burden of stroke in this patient population.

## 1. Introduction

Stroke is a leading cause of mortality and disability globally and places a significant economic burden on patients and healthcare systems. Over the past 20 years, the treatment of acute ischemic stroke (AIS) has progressed with the advent of intravenous thrombolysis and mechanical thrombectomy (MT).^[1–6]^ Nevertheless, acute stroke reperfusion therapies rely greatly on prompt intervention and advanced facilities, and, therefore, they cannot mitigate the consequence of the stroke in a substantial proportion of patients.^[7,8]^

There is a vast amount of literature available on the burden of AIS. The majority of available evidence published in existing systematic literature reviews (SLRs) relates to overall AIS populations with a general focus on mild cases (<5 National Institute of Health Stroke Scale [NIHSS]),^[9–11]^ potentially due to the fact that more than half of all AIS events are classified as mild or transient in nature.^[12]^ However, given the importance of stroke severity on patient outcomes, it is desirable to aggregate where available data regarding the burden of more severe AIS. Although studies of the clinical burden of AIS are typically stratified by severity, other aspects of disease burden, such as humanistic and economic, are not. The authors of this study aimed to conduct an SLR with 2 distinct components: a review of clinical burden studies (including epidemiology) with a specific focus on patients with moderate to severe AIS; and a review of economic burden and humanistic burden, including a review of economic evaluations looking at the overall AIS population to present a holistic overview of the contemporary disease burden considering the rapidly changing nature of stroke care.

## 2. Materials and methods

An SLR was conducted in line with the Cochrane handbook for SLRs and reported in line with the 2020 Preferred Reporting Items for Systematic Reviews and Meta-Analyses statement.^[13,14]^ Searches were run on June 21, 2023, using the OVID® platform across the Embase, MEDLINE®, EconLit, PsycINFO, and Evidence-Based Medicine Reviews databases (refer to Tables S1–S10, Supplemental Digital Content, http://links.lww.com/MD/O276). Studies written in English language, and published between January 1, 2015, and June 21, 2023, were reviewed. Studies were limited to 2015 onwards, reflecting an important breakthrough year for the treatment of stroke, as 5 clinical trials published in the peer-reviewed literature demonstrated the positive effect of MT on patients with AIS compared to medical therapy alone.^[2–6]^ The search strategies were tailored to the 5 OVID® databases, including specific key terms (eg, acute ischemic stroke or hospitalization in Embase) in combination with free-text searching (multipurpose terms) and subject headings for the clinical, economic, and humanistic burden of AIS as well as terms for study designs and countries of interest.

The inclusion and exclusion criteria for screening are outlined in the supplementary information; these were based on the Population, Intervention, Comparators, Outcomes, Time horizon, Study design framework outlined in Table S11, Supplemental Digital Content, http://links.lww.com/MD/O276. In this SLR, burden is defined as the overall impact that a disease has on the individual as measured by the sum of the clinical, humanistic, and economic burden on individuals. The focus for the clinical burden outcomes was limited to those with moderate to severe AIS from the screening stage, specifically excluding mild AIS (NIHSS ≤ 4) populations.^[15]^ For the other outcome categories, such as humanistic burden (defined as the impact of the disease on the individual quality-of-life [QoL] and activities of daily living),^[16,17]^ economic burden, and economic evaluations, the overall AIS population was considered regardless of severity status after an initial search trying to focus on more severe strokes revealed a lack of studies. Abstracts and full-text screening were conducted independently by 2 reviewers, blinded to each other’s decisions. In instances where there were review conflicts, a third reviewer was consulted to resolve the conflicts. Extractions were completed by 1 reviewer and quality checked by a senior reviewer. Data from the included studies were extracted into a bespoke data extraction form (DEF) utilizing Microsoft Excel; this DEF was made to suit the purpose of this SLR and the study types that were included. The DEF included multiple sheets to account for variations in reporting of outcomes (eg, observational studies vs economic evaluations). The Joanna Briggs Institute (JBI) Handbook for Evidence Synthesis was utilized to assess risk of bias; this was completed by 1 reviewer and verified by a senior reviewer.^[18]^ We utilized the JBI critical appraisal tools, which include multiple checklists that are tailored towards the evidence type and study designs that we expected to encounter in this project and cover cohort, cross-sectional, other observational, and economic evaluation study designs. For economic studies reporting cost data in local currencies, cost conversions into US Dollars (USD; 2023) were performed using the cost year of the reference study and Organization for Economic Co-operation and Development exchange rate data, along with Federal Reserve Economic Data inflation rates to account for changes in purchasing power over time.^[19]^

## 3. Results

**Figure 1.**
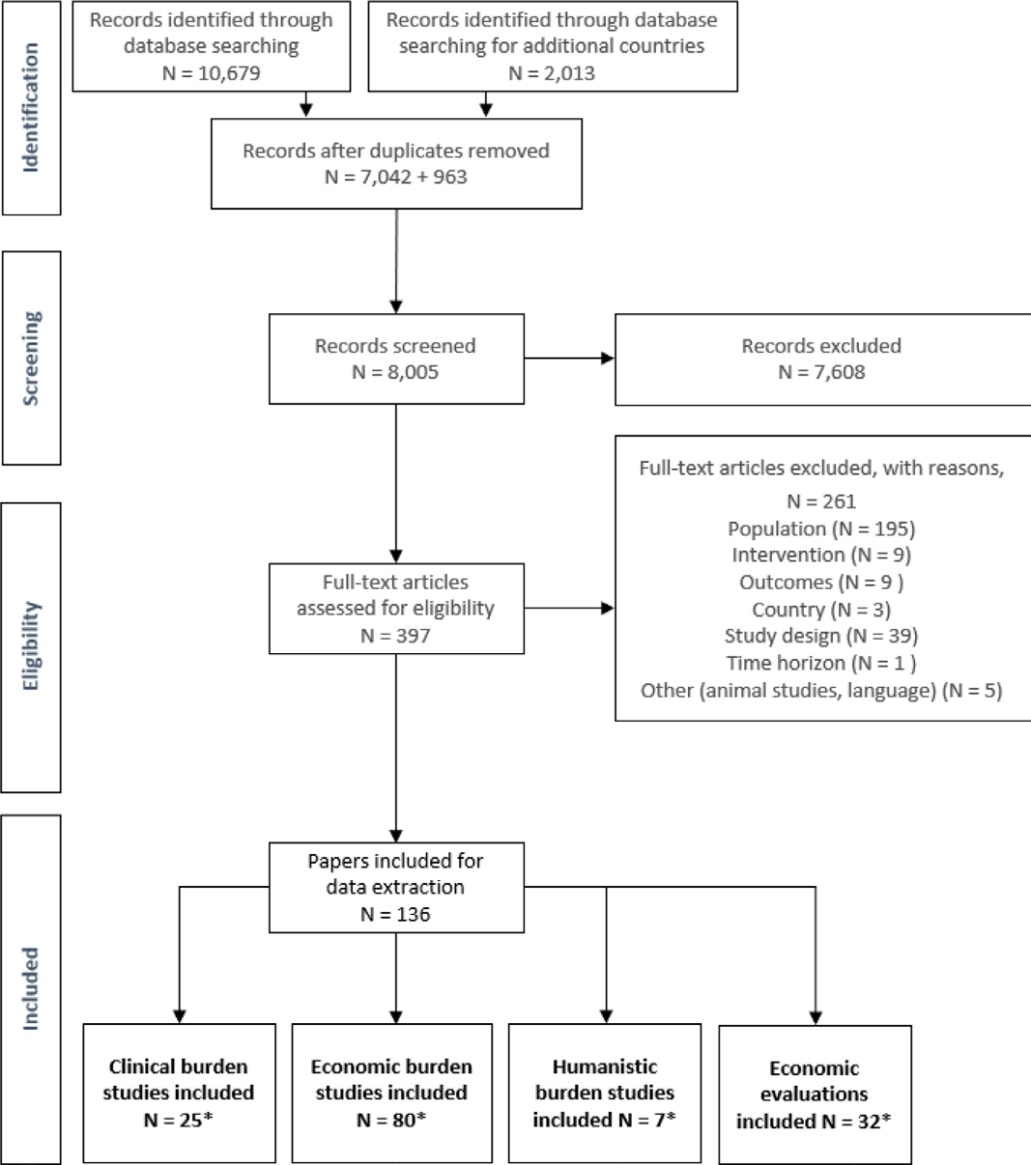
Preferred Reporting Items for Systematic Reviews and Meta-Analyses diagram. *The number of studies across outcome types add up to >136 as some publications may report on more than 1 outcome type.

Overall, the database searches identified 12,692 records; however, once duplicates were removed, 8005 records were screened, with 136 publications meeting the study entry criteria (Fig. 1). Across these publications, 25 reported on the clinical burden relating to moderate to severe AIS. In addition, 7 focused on the humanistic burden, 80 on the economic burden, and 32 reported economic evaluations in AIS overall.

### 3.1. Clinical burden

Across the 25 studies reporting on the clinical burden of moderate to severe AIS, the most commonly reported outcomes were mortality (n = 16), functional outcomes (n = 12), and stroke recurrence (n = 4). Moderate to severe AIS was associated with a high mortality rate, which increased with disease severity (Table S12, Supplemental Digital Content, http://links.lww.com/MD/O276). Where studies reported mortality rates among patients with moderate AIS (NIHSS 5–15), these ranged from 6.5% in-hospital mortality over 36 months (median [interquartile range] length of stay [LOS] 9 [14] days) in patients with large vessel occlusions in Saudi Arabia^[20]^ to 36.8% all-cause mortality (after 36 months) in patients with large-artery atherosclerosis (LAA) from China.^[21]^ Seven studies specifically reported mortality rates among patients with moderate to severe AIS (NIHSS ≥ 15) with the mortality rates ranging from 9.9% 90-day mortality in male patients with severe AIS from Japan,^[22]^ to 67.6% in patients after 36 months with severe AIS attributable to a LAA from China.^[21]^ These data highlight the high levels of variability relating to mortality; however, additional information with a formal comparison to mild stroke is needed before the drivers of the variability can be ascertained.

Studies that examined the effect of AIS severity on functional outcomes indicate that increased stroke severity at presentation is associated with increased levels of poststroke disability. For instance, Wu et al (2021) reported that in China, patients with severe (reported as NIHSS ≥ 17) compared to moderate AIS (reported as NIHSS 8–16) had higher rates of dependency or death defined as a modified Rankin Scale (mRS) > 2, at 3 months (18.6% vs 17.1%), 12 months (44.9% vs 41.6%), and 36 months (100% vs 86.6%) post stroke onset.^[21]^ The SLR also identified a number of studies that compared the level of disability among patients successfully treated and not successfully treated with reperfusion therapies in the real-world setting. Where reported, studies highlighted the considerable benefit of reperfusion treatment on the level of disability poststroke. For example, Mueller-Kronast et al (2017) highlighted that the rate of good functional outcomes (mRS 0–2) at 90 days was significantly higher in patients with successful reperfusion compared to those that did not achieve reperfusion (60.0% vs 40.4%; *P* < .001), indicating that successful treatment is associated with reduced disability following AIS.^[23]^ These studies underline a significant unmet need in patients who do not receive reperfusion therapy or who are not successfully reperfused.

**Figure 2.**
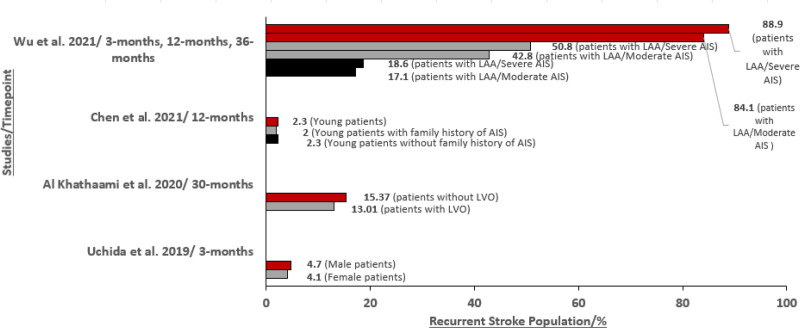
Stroke recurrence in untreated patients. AIS = acute ischemic stroke, LAA = large-artery atherosclerosis, LVO = large vessel occlusion.

Four studies reported stroke recurrence outcomes among patients with moderate-severe stroke with studies predominately being identified in Asia (n = 4), with 2 in China and 1 each in Saudi Arabia and Taiwan. Overall, these studies highlight that the rate of stroke recurrence varied depending on the length of follow-up, but initial stroke severity is associated with greater risk of stroke recurrence across studies (Fig. 2). For instance, Wu et al (2021) reported that Chinese patients aged 60 years and older with severe LAA AIS (baseline NIHSS ≥ 17) had a higher cumulative stroke recurrence rate compared to patients with moderate to severe LAA AIS (baseline NIHSS 8–16) at 3 months (18.6% vs 17.1%), 12 months (50.8% vs 42.8%), and 36 months (88.9% vs 84.1%).^[21]^ Whereas in Uchida et al (2019), the 3-month recurrence rate was 4.7% in males and 4.1% in females (compared to the relatively high values presented in Wu et al [2021]). Interestingly, in the Uchida et al (2019) study, the severity of initial stroke was the only stroke characteristic found to have an impact on the rate of stroke recurrence following index AIS.^[21]^ Other factors that were investigated in identified studies but were found to have no significant impact on the incidence of stroke recurrence were sex,^[22]^ stroke characteristics including the presence of large vessel occlusions,^[20]^ and familial history of AIS.^[24]^

Of the identified studies, all were considered to have a low risk of bias when critically appraised using the JBI tools except for 3 studies that had a moderate risk of bias (due to the reporting of study methods); therefore, the clinical burden studies were generally of good quality with reliable outcomes. Overall, moderate to severe AIS places a substantial clinical burden on patients, with high mortality and recurrence rates, which increase with disease severity. Severe AIS events are also associated with worse functional outcomes compared to moderate events. Furthermore, patients who do not receive successful reperfusion treatment have worse functional outcomes compared to successfully treated patients, suggesting a significant unmet need for patients who don’t receive or don’t respond to reperfusion treatment.

### 3.2. Humanistic burden

Limited humanistic burden data relating to patients with AIS were identified in this SLR regardless of disease severity. Five studies identified patients with AIS using the International Classification of Diseases, 10th Revision, primary discharge diagnosis codes,^[25–29]^ while 2 used radiographic diagnosis methods.^[30,31]^ Data comprising patient characteristics, quality indicators, and health outcome measures were sourced from hospital systems, and long-term outcomes were obtained centrally by survey methods (eg, for health-related quality-of-life [HRQoL]) or annual data linkage to national death registrations.^[25–29]^ The most widely reported tools used to measure humanistic burden were the European Quality-of-Life 5 Dimensions 3 Level Version,^[30]^ the Patient Reported Outcomes Measurement Information System,^[26]^ the Montreal Cognitive Assessment,^[28]^ the Barthel Index,^[28]^ the Hamilton Rating Scale for Depression,^[31]^ the Korean mini-mental state examination,^[29]^ and the Quality-of-Life in Neurological Disorders Executive Function Scale.^[26]^ Four studies reported on patient reported outcomes with 3 studies conducted in the United States^[25–27]^ and 1 in South Korea.^[29]^ A United States-based study utilized the Patient Reported Outcomes Measurement Information System tool and reported the most commonly reported patient reported outcome domains in patients with AIS were physical functioning (mean [standard deviation] 58.8 [10.7]) and satisfaction with social roles (mean [standard deviation] 585.4 [11.3]).^[26]^ Additionally, no studies addressed caregiver burden and the multifaceted strain exerted on caregivers from caring for an individual following AIS.^[32]^

Where reported, cognitive impairment was associated with worse HRQoL across all domains, that is, physical activity restriction, severity of pain, and social activity restriction (Table S13, Supplemental Digital Content, http://links.lww.com/MD/O276).^[25,28,29]^ Female sex and the presence of certain comorbidities (such as dysphagia) were both identified as risk factors for cognitive impairment among patients with AIS.^[25,28,29]^ In the United States, a cross-sectional study of older patients with AIS and comorbid dysphagia on Medicare (mean age of 78.7 years) exhibited significantly higher rates of cognitive impairment compared to patients without dysphagia (29.2% vs 18.9%; *P* < .001).^[25]^ While a prospective cohort study in China found that female patients with AIS exhibited higher rates of cognitive impairment (86.8% vs 62.8%) and physical dependence (66.8% vs 58.5%) compared to male patients over a 1-year period, in addition to lower EQ-5D index scores (0.73 vs 0.76) and EQ-5D visual analogue scale scores (71.9 vs 73.1).^[28]^

Four of the 7 studies reported a positive correlation between patients with AIS and poststroke depression (PSD) (Table S13, Supplemental Digital Content, http://links.lww.com/MD/O276).^[25–27,31]^ Three United States-based studies reported that patients with AIS had high rates of PSD,^[25–27]^ with 1 study also highlighting that patients with AIS had a 50% increased likelihood of developing depression during a 1.5-year follow-up when compared to depression in patients postmyocardial infarction.^[27]^ High rates of depression were also reported in China by Wang et al (2017) at 2 weeks (25.4%), 3 months (17.6%), and 12 months (12.4%) poststroke.^[31]^ Across all studies, PSD was consistently associated with lower HRQoL and self-reported health status, with female patients found to have worse overall HRQoL compared to male patients’ poststroke.^[25–27,31]^ A sex disparity between males and females in poststroke recovery was also reported, whereby physical dependence (Barthel index < 95) in female patients with AIS was significantly greater than in male patients (66.8% vs 58.5%; *P* < .001).^[28]^ Overall, patients with AIS reported neuro-QoL executive function scores of 45.6%, which were significantly lower than the general population, indicating marked worsening in executive function following stroke.^[26]^

### 3.3. Economic burden

Eighty studies were included that examined the economic burden associated with AIS across 19 countries, with the largest source of data derived from US commercial insurance claims databases, Medicare and Medicaid.^[33–61]^ Outside of the United States, the majority of data were sourced from national registries, hospital databases, and stroke cohort studies.

**Figure 3.**
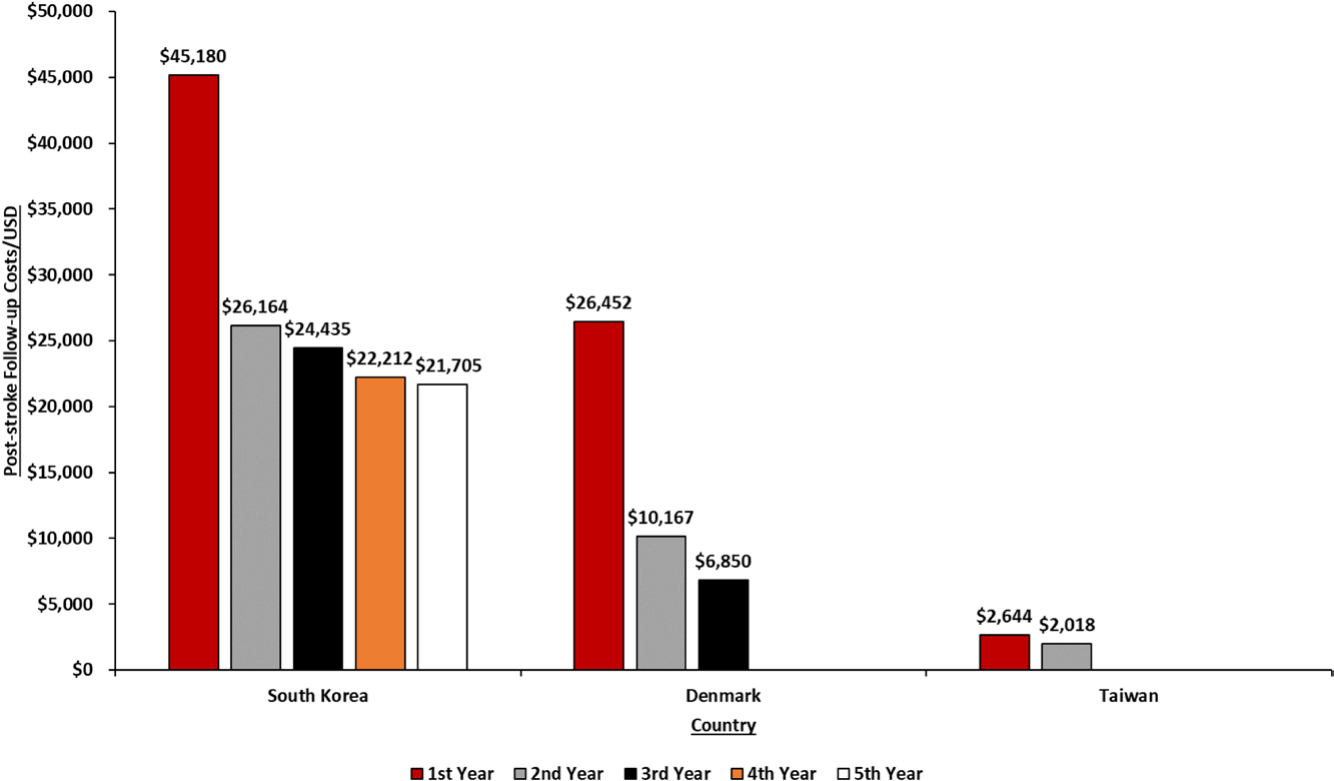
Poststroke follow-up costs across included studies (inflated to 2023 USD values).

The most frequently reported measures were total costs and hospital LOS associated with AIS treatment and care. Overall, the cumulative healthcare costs associated with AIS were high, incurring substantial cumulative inflation-adjusted costs, amounting to approximately $195.9 billion USD (2023) among 9,009,007 patients in the United States, from 2002 to 2017.^[40]^ Twenty-three studies analyzed the cumulative costs of AIS. A study by Yousufuddin et al. (2020) stratified results by patient subgroups such as age, sex, race, and comorbidity status. The highest monthly mean costs up to 3-years poststroke in the United States were associated with female sex ($6214), older patients (≥85 years old–$6823), and non-Caucasian individuals ($8036).^[46]^ This large economic burden imposed by AIS on healthcare systems is a global trend as observed in Russia, where patients with AIS and comorbid diabetes mellitus were associated with high cumulative costs amounting to ₽7.0 billion Russian ruble in 2014 (USD 2023 conversion $230.1 million).^[62]^ Additionally, multiple studies reported on the costs associated with AIS across multiple time points with high initial costs poststroke; however, these costs were seen to decrease with time as demonstrated in Figure 3.^[63–65]^ The cumulative healthcare costs associated with AIS were high in Finland, particularly when compared to other neurocritical illnesses including traumatic brain injury, intracerebral hemorrhage, and subarachnoid hemorrhage, amounting to €39,222 euros (EUR [2013]; [USD 2023 conversion $67,274]) per patient.^[66]^ As previously outlined, there are limited data relating to the economic burden associated specifically with moderate to severe AIS. However, 1 study investigated the effect of stroke severity on hospitalization costs and reported that severity was strongly associated with costs. For instance, compared to patients with an NIHSS score of 0–5 ($2803 USD 2023), those with a score of 6–10 incurred 58% higher cumulative monthly costs ($5132 USD 2023) while those with an NIHSS score of 11–15 faced 329% greater costs ($15,487 USD 2023).^[46]^

**Figure 4.**
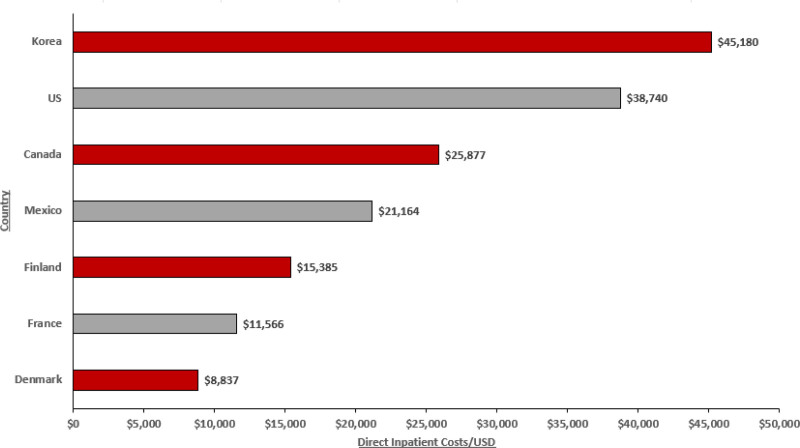
Inpatient costs by country (adjusted to 2023 USD values).

Where reported, studies documented high inpatient costs for patients with AIS, with higher direct costs correlating with hospital transfers, stroke center admission, and readmission (Table S14, Supplemental Digital Content, http://links.lww.com/MD/O276). Data were identified from 8 countries, including the United States (n = 20), China (n = 7), Denmark (n = 2), Taiwan (n = 1), South Korea (n = 1), Mexico (n = 1), Finland (n = 1), and France (n = 1). An overall breakdown of total inpatient costs inflated to 2023 USD values by country is reported in Figure 4, with the highest direct inpatient costs reported in South Korea ($45,180 USD) and the United States ($38,740 USD). Lower total inpatient costs were reported in countries such as Denmark ($8837 USD), France ($11,566 USD), and Finland ($15,385 USD), with this likely being due to differences in the healthcare system structure across these countries. Of particular note, in the United States, patients with AIS accrued substantial inpatient hospitalization costs, averaging approximately $38,740 USD per patient.^[54]^ Furthermore, higher costs were incurred by admission to a primary stroke center ($47,621 USD) compared to admission to a nonprimary stroke center ($35,229 USD).^[54]^ Likewise, higher costs of hospitalization, including all patients who had an intervention, were sustained by patients with AIS transferred from another facility ($97,546 USD 2023) than those directly admitted ($70,325 USD 2023).^[45]^ Furthermore, Sonig et al (2016) also highlighted that hospital costs were significantly higher for patients transferred from other facilities ($119,890 USD [2023]) compared to direct admissions ($86,433 USD [2023]), with transferred patients also having a higher rate of discharge to other healthcare facilities rather than being discharged home, highlighting the importance of receiving appropriate stroke care from the onset of stroke.^[45]^

A limited number of studies (n = 3) reported on the indirect outpatient costs associated with AIS, including productivity losses and forgone earnings due to inability to work.^[67–69]^ Jennum et al (2015) estimated the indirect costs associated with productivity loss in Denmark over 3 years attributable to AIS in patients with atrial fibrillation at $2873 USD (2023).^[68]^ The study also estimated the indirect costs (loss of labor market income) for caregivers at $1618 USD (2023). In Sweden, the highest indirect costs associated with AIS were reflected in patients with hyperlipidemia, amounting to €6784 Euro (EUR; 2016) (USD 2023 conversion $8896).^[69]^

**Figure 5.**
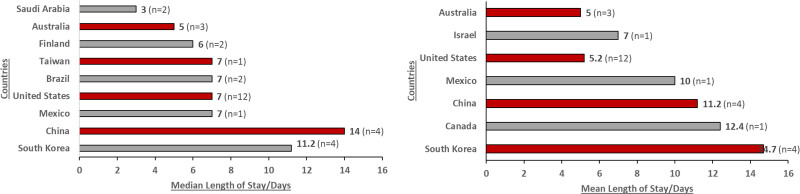
Length of stay by country (median and mean values).

AIS imposes an extensive economic burden on healthcare systems globally due to increased resource use, extended LOS, and high admission and readmission rates (Fig. 5). In the United States, the median LOS for patients with moderate-to-severe AIS was 7 days.^[50,51]^ These findings were consistent with Mexico, Brazil, Israel, and Taiwan (mean LOS was 7 days).^[70–73]^ Patients with comorbidities or cardiac complications tended to have increased resource use compared to patients without either, with 2 studies reporting a correlation between patients with AIS and cardiac complications and an extended LOS.^[33,74]^ Iguchi et al (2021) reported that across 19 hospitals in Japan, patients with AIS and heart failure were associated with higher in-hospital LOS (30 days);^[74]^ Aggarwal et al (2021) reported that patients with AIS and concurrent MI in the United States had longer LOS, higher hospitalization costs, and less frequent discharges to home compared to admissions for acute MI alone.^[33]^ When assessing the transfer rate to a rehabilitation facility in the United States based on type of health insurance, patients with Medicare had the highest transfer rate (32.7%), followed by Medicaid (29.3%) and private insurance (25.1%).^[44]^ Uninsured patients had a reported transfer rate of 18.8% to rehabilitation facilities poststroke, which was associated with a 40%–50% lower odds of being transferred compared to patients with insurance coverage.^[44]^ In addition, they were twice as likely to be discharged home poststroke.^[44]^ Findings show that hospital transfer rates in countries such as the United States may be more associated with patients’ insurance coverage rather than their disease status and clinical needs at the time of hospital discharge.^[44]^

### 3.4. Economic evaluations

Thirty-two economic evaluations assessing AIS treatments were identified in this SLR. The 17 countries covered included the United States (n = 6), China (n = 5), the United Kingdom (n = 4), Australia (n = 2), Canada (n = 2), Spain (n = 2), Argentina (n = 1), France (n = 1), Indonesia (n = 1), Italy (n = 1), Japan (n = 1), Norway (n = 1), Saudi Arabia (n = 1), Sweden (n = 1), and multiple countries (n = 3). The majority of studies utilized a Markov stage transition model approach. Across studies, the most frequently reported health states used correlated with the 7 mutually exclusive health states based on the mRS (0=no symptoms, 6=death; n = 14) or mRS health states as trichotomous variables (n = 10) 0–2 (good functional outcomes), 3–5 (poor functional outcomes), and 6 (death). The most frequently used time horizons were lifetime (n = 9) and 5 years (n = 8). The majority of models adopted a payer/healthcare perspective (n = 27) with 5 studies adopting a societal perspective (three in the United States and 1 each in Sweden and Saudi Arabia, respectively).^[75–79]^ The discount rate utilized across studies varied, ranging between 2% and 5%, with most studies using a 3%–3.5% discount rate (n = 20). The most frequently reported interventions were MT (n = 14), followed by thrombolytics (n = 8), and combination MT and thrombolytics (n = 7). The most reported comparators were standard of care (SoC) (n = 9), followed by thrombolytics (n = 8). Overall, the SLR identified that MT is cost-effective versus SoC alone or in combination with thrombolytics. For instance, Xie et al (2016) developed a Markov model assessing the cost-effectiveness of MT with intravenous thrombolytics versus treatment with intravenous thrombolytics alone from the public payer perspective in Canada. The study reported that MT in combination with thrombolytics was cost-effective (cost: $126,939 990 Canadian dollars [CAD], 1.484 quality adjusted life years [QALYs]) compared with treatment with intravenous thrombolysis alone (cost: $124,419 USD and 1.273 QALYs) with an incremental cost-effectiveness ratio (ICER) of $11,990 CAD per QALY gained.^[80]^ Kabore et al (2019) also reported that MT in combination with intravenous thrombolytics was a cost-effective treatment (€14,715 per QALY gained) compared to intravenous thrombolytics alone. Additionally, the cost-effectiveness of MT versus SoC was assessed across 2 studies.^[81,82]^ Sanmartin et al. (2022) conducted an economic analysis combining a decision tree and Markov model to estimate in the United States the lifetime costs and QALYs associated with the use of MT compared with SoC (not defined in the study) in patients with AIS. Overall, the study reported that MT was cost-effective versus SoC with higher lifetime benefits (2.20 QALYs vs 1.41 QALYs), even though it produced higher lifetime healthcare costs per patient ($285,861 USD vs $272,954 USD),^[81]^ and better outcomes with an ICER of $16 239/QALY.^[81]^ Kunz et al (2016) utilized a similar model structure to Sanmartin et al (2022) and examined the cost-effectiveness of MT with SoC versus SoC alone in the United States using a decision tree approach based on Markov simulations (SoC was not defined). Overall, MT plus SoC was cost-effective compared with SoC alone (incremental cost: $4938 USD, incremental effectiveness: 1.59 QALYs, and ICER: $3110/QALY).^[82]^

## 4. Discussion

Published literature available on AIS is vast; however, this is one of the first SLRs focusing on the clinical burden of patients with moderate to severe AIS while additionally examining the economic burden, humanistic burden, and a review of economic evaluations in the overall AIS population.

Studies reported mortality rates of up to 36.8% in patients with moderate AIS^[21]^ and 67.6% in patients with severe LAA AIS,^[21]^ highlighting the considerable fatality burden of AIS. The World Stroke Organization estimate of 42.6% mortality for all AIS in 2019,^[83]^ therefore, understates the clinical burden found in the more severe stroke population as evidenced by the findings reported in this SLR. This means that countries worldwide should implement further AIS prevention and treatment measures as part of their healthcare policy to reduce the mortality from AIS. Interestingly, the SLR only identified a small number of studies reporting on stroke recurrence in moderate-severe AIS patients, with a large variation in the rate of recurrence across studies. This variation could relate to a multitude of factors (eg, age and gender). However, only greater severity of stroke was found to have an impact on the rate of stroke recurrence following index AIS.

Among patients with AIS, the most affected HRQoL domain was physical function, closely followed by satisfaction with social roles and executive function. Impairments in physical function are a common feature of stroke and are a key focus of rehabilitation efforts. However, social participation and cognitive functioning have not received as much attention. Understanding the extent of impairment in these domains is crucial for optimizing patient well-being and implementing appropriate interventions. Patients often report significant cognitive decline and PSD, both of which are associated with decreased HRQoL and worse self-rated health across all domains. Additionally, patients with AIS and poststroke dysphagia experience worse HRQoL compared to those without poststroke complications. Given that dysphagia affects more than 50% of stroke survivors, with 11%–13% remaining dysphagic after 6 months, there is a large burden imposed on these patients.^[84]^ Recognizing the association between PSD and poststroke dysphagia is important to understand the added burden in this patient population. Early identification through targeted and repeated depression screenings during the rehabilitation period can facilitate timely referrals for comprehensive evaluation and treatment of depression as needed and should be implemented in healthcare policy globally. Separately, female patients with AIS generally exhibited worse HRQoL compared to male patients, underscoring the need for further research to understand the significance of sex as a biological variable in AIS studies.^[85]^ Despite advancements in investigating sex disparities in stroke and factors affecting risk and outcomes in females, notable research gaps persist.^[85]^ Often, sex and gender have been combined in stroke research, and continued efforts are needed to disentangle the impact of biological sex from the social implications of gender-related factors. While several risk factors specific to females have been identified, current stroke prediction models do not integrate these female-specific risk factors.^[86]^ Given the older age and greater disability among women prestroke and their worse poststroke outcomes, understanding the role of access to clinical care, rehabilitation services, and sociodemographic factors influencing these sex disparities is imperative.

AIS has a substantial economic impact globally, encompassing high inpatient and outpatient costs, further demonstrating the need for worldwide healthcare policy changes to the treatment and management of AIS to reduce the economic burden. This SLR presents a comprehensive overview of total healthcare costs in patients with AIS. The findings of this SLR confirm that patient costs are higher in high-income countries, consistent with previous research.^[87]^ However, few studies have explored outpatient costs, indirect costs associated with AIS discharge, productivity losses, foregone earnings, and ongoing medical expenses required for risk management and poststroke rehabilitation. Indirect costs due to lost productivity account for an important part of the total costs of cardiovascular disease.^[69]^ The American Heart Association estimated that in the United States, in 2023, direct and indirect costs of total cardiovascular disease were $448.7 billion, and that cardiovascular disease-related indirect costs were US $171.7 billion in 2023. At the same time, patients with AIS and comorbidities incur a greater economic burden compared to those without comorbidities. Economic data related to caregiver burden were limited with only 1 study highlighting the economic burden experienced by caregivers. Spouses of care recipients often constitute the highest number of caregivers, and women represent the largest portion of this group.^[88]^ Further research into the burden of caregiving for patients with AIS is necessary to improve the health and well-being of family caregivers and guide policymakers in developing practices to maintain the health of the caregiver population.^[88]^ Additionally, the heterogeneous data aligns with previous research findings, suggesting that variations in level of care, medical resources, study type, geography, and sample size may contribute to differences in LOS and cumulative costs. For example, study populations and timeframes potentially having an effect on findings; for instance, 2 studies had relatively similar findings with Jakobsen et al (2016) exploring AIS costs up to 3 years from first stroke in the general Danish population covering the timeframe from 2002 to 2012 and Kim et al (2020) examining costs up to 5 years in AIS patients between 2011 and 2016 in South Korea. These studies differed from Lin et al (2021), who reported on patients with AIS who went undiagnosed for up to 3 years after their initial stroke as identified through the Taiwanese National Health Insurance Research Database between 2011 and 2015; the difference in the study population could have led to the substantially different results over time compared to Jakobsen et al (2016) and Kim et al (2020).

Longer LOS often signifies a more severe condition and complex treatment requirements. However, it also implies that these seriously ill patients receive more medical resources and sustained, attentive care, which can lead to improved outcomes. Conversely, LOS is often difficult to interpret for AIS due to the high risk of death or being transferred to another care facility. In these cases, the LOS figure is skewed with a negative outcome but results in a positive (smaller) LOS value. Further analyses are warranted to assess cost estimates at each stage of the AIS care continuum^[87]^ to facilitate the development of more effective stroke-related financing policies, thus improving outcomes in these patients.

A major strength of this SLR was the comprehensive search of literature across 5 different databases in line with the Cochrane guidelines and Preferred Reporting Items for Systematic Reviews and Meta-Analyses statement best practices to ensure all high qualitative relevant literature was covered. As stroke is a well-published disease area with extensive literature available, an additional strength of the SLR is the broad global coverage. The SLR included 28 countries, including the G20 countries, Scandinavia, and Israel; therefore, countries from all continents are covered. From an economic burden perspective, the SLR inflated costs to 2023 USD values, allowing for a direct comparison of costs over different periods and countries, providing a more consistent and accurate analysis of cost trends and differences. Nevertheless, this SLR is subject to some limitations. Conference abstracts were excluded from the review, which means that the most recent available data may not be captured. It should be noted, however, that abstracts are not peer-reviewed and present incomplete and preliminary findings, methods, and details. Furthermore, this SLR was limited in making direct comparisons within the data due to the large heterogeneity between studies. This heterogeneity was present across populations, with large differences in patient characteristics across studies included in this SLR and in the reporting of outcomes, with different comparators, time points, and unit types (eg, hazard ratios, odds ratios, and discrete values) being used across studies. With regard to the inflation of costs to 2023 USD values, there is a potential limitation due to the difficulty of obtaining precise historical inflation data for different countries, with the potential for discrepancies which can lead to inaccurate adjustments. However, the Organization for Economic Co-operation and Development and Federal Reserve Economic Data are reputable sources for inflation rates that should limit the potential for discrepancies. Although not necessarily a limitation of this SLR, 1 final consideration relates to the exclusion of RCTs from the eligibility criteria given their importance in understanding treatment efficacy. However, it was deemed acceptable to exclude these study types as they primarily provide information on treatment-related outcomes within a controlled setting and highly selected populations and do not reflect care provided in clinical practice and the burden of disease or cost of care.

The findings from this SLR highlight the high disease burden associated with AIS, specifically for patients with moderate and severe disease, as demonstrated by the increased mortality and disability that comes with increasing stroke severity. The humanistic burden of moderate and severe stroke remains underreported, with few studies investigating symptoms and impacts on patients’ HRQoL or sex disparities in functional outcomes and no studies reporting the caregiver humanistic burden with only 1 study identified in the SLR reporting on caregiver economic burden. The SLR also highlights that AIS imposes a significant economic burden on healthcare systems, mostly driven by high hospitalization costs. Going forward, additional research focused on outcomes after moderate to severe AIS is needed to fully understand the overall disease burden on these patients.

## Acknowledgments

Medical writing and editorial assistance were provided by Ashley Enstone and Peter O’Donovan of Adelphi Values PROVE™, Bollington, Cheshire, UK. This assistance was funded by Shionogi Inc., NJ, a subsidiary of Shionogi & Company, Osaka, Japan. The listed authors have authorized the submission of their manuscript via third party and approved any statements or declarations.

## Author contributions

**Conceptualization:** Terry Quinn, Kazuo Kitagawa, Thomas Leung, Carlos Molina, Alejandro Rabinstein, Roy Bentley, Owen Henry, Maria Heuser, Vedes Nair, Jeffrey Saver.

**Supervision:** Terry Quinn, Kazuo Kitagawa, Thomas Leung, Carlos Molina, Alejandro Rabinstein, Roy Bentley, Jeffrey Saver.

**Data curation:** Owen Henry, Maria Heuser, Vedes Nair.

## Supplementary Material


