# Supplementary material for: A Systematic Literature Review on the Burden of Disease for Patients With Moderate to Severe Acute Ischemic Stroke
Source: Medicine (Baltimore). 2025 Jan 17;104(3):e41249. doi: 10.1097/MD.0000000000041249 (PMC11749521; doi:10.1097/MD.0000000000041249)
Supplement: Supplementary file 1 [file medi-104-e41249-s001.docx]

**Table 1 Embase Search Strategy**

Database: **Embase**1974 to 2023 June 19^th^
Search Strategy: June 21^st^, 2023

| # | Searches | Results |
| --- | --- | --- |
| 1 | *acute ischemic stroke/ | 6011 |
| 2 | (isch?emic adj3 stroke).tw. | 134362 |
| 3 | or/1-2 | 134589 |
| 4 | *hospitalization/ or *hospital patient/ or *emergency ward/ or *"length of stay"/ | 142385 |
| 5 | *morbidity/ or *mortality/ | 137650 |
| 6 | (morbidity or mortality).ti,ab. | 1716837 |
| 7 | *social care/ or *home care/ or *long term care/ or *nursing home/ or *elderly care/ | 111468 |
| 8 | ((social or home or elderly or follow*) adj3 care).ti,ab. | 108573 |
| 9 | (hospitali?ation or outpatient or inpatient or (length adj5 stay) or (emergency adj3 (ward or department))).ti,ab. | 984707 |
| 10 | (clinical adj5 (burden or impact)).ti,ab. | 92961 |
| 11 | or/4-10 | 2761449 |
| 12 | *incidence/ or *prevalence/ or rankin scale/ | 168332 |
| 13 | (incidence or prevalence or "modified rankin scale").ti,ab. | 2365339 |
| 14 | 12 or 13 | 2380984 |
| 15 | *"quality of life"/ or *quality adjusted life year/ or *disability-adjusted life year/ or *"European Quality of Life 5 Dimensions questionnaire"/ | 142867 |
| 16 | (eq adj3 5d).mp. or ("STROKE-QoL" or "SS-QoL" or "QLASS").ti,ab. | 23715 |
| 17 | (quality adj5 life).ti,ab. | 611281 |
| 18 | or/15-17 | 629811 |
| 19 | (*Health Care Economics/ and Organizations/) or *"cost of illness"/ or *"hospital cost"/ or *"hospitalization cost"/ or *"cost control"/ or *"drug cost"/ or *"health care cost"/ or *health care utilization/ or *productivity/ or medical leave/ | 119406 |
| 20 | *economic evaluation/ or *"cost benefit analysis"/ or *"cost effectiveness analysis"/ or *"cost utility analysis"/ or *cost minimization analysis/ | 61862 |
| 21 | (Cost* or expen* or financ* or price* or pricing or ((economic or societ* or socioeconomic or socio economic or illness or disease or patient* or caregiver* or carer*) adj2 burden)).ti,ab. | 1471941 |
| 22 | ((resource* adj2 (utili?ation or use*)) or productivity or hospitali?ation* or (leave adj2 (medical or sick or disability))).ti,ab. | 525076 |
| 23 | (cost adj (effective* or utilit* or minimi* or benefit)).ti,ab. | 255203 |
| 24 | or/19-23 | 1925241 |
| 25 | *clinical practice/ | 53642 |
| 26 | exp *practice guideline/ | 125484 |
| 27 | *unmet medical need/ | 566 |
| 28 | ((clinical or treatment or therap*) adj5 (practice* or pattern* or trend* or guideline* or recommend* or barrier* or challenge*)).tw. | 905940 |
| 29 | ((unmet or me?t*) adj3 (need? or demand? or require*)).tw. | 281016 |
| 30 | or/25-29 | 1286061 |
| 31 | 3 and (11 or 14 or 18 or 24 or 30) | 64232 |
| 32 | United States/ or Japan/ or France/ or Germany/ or Italy/ or Spain/ or United Kingdom/ or China/ or Wales/ or Scotland/ or England/ or Northern Ireland/ or Australia/ or Sweden/ or Denmark/ or Canada/ or South Korea/ or Norway/ or Taiwan/ | 3165186 |
| 33 | (US or USA or (United adj States) or Japan or France or Germany or Italy or Spain or China or UK or (United adj Kingdom) or England or Wales or Scotland or Northern Ireland or Australia or Sweden or Denmark or Canada or South Korea or Norway or Taiwan).ti,ab. | 3116135 |
| 34 | or/32-33 | 4788284 |
| 35 | 31 and 34 | 12869 |
| 36 | limit 35 to yr="2015 -Current" | 8255 |
| 37 | exp conference paper/ or conference abstract/ or (conference adj (abstract or paper or review or proceeding)).pt. | 5595651 |
| 38 | limit 37 to yr="2015 - 2019" | 1976333 |
| 39 | 36 not 38 | 6135 |
| 40 | exp case study/ or case report/ or letter/ or editorial/ or note/ | 5538816 |
| 41 | (editorial or letter or case report or comment or news).pt. | 2089138 |
| 42 | 39 not (40 or 41) | 5960 |
| 43 | (exp animal/ or exp invertebrate/ or nonhuman/ or animal experiment/ or animal tissue/ or animal model/ or exp plant/ or exp fungus/) not (exp human/ or human tissue/) | 7740885 |
| 44 | 42 not 43 | 5917 |
| 45 | Clinical Study/ or Case-Control Studies/ or Longitudinal Studies/ or Prospective Studies/ or Cohort Studies/ or Observational Study/ or Cross-Sectional Studies/ or Register/ | 2580648 |
| 46 | ((Cohort or "case control" or "follow up" or observational or "cross sectional" or "regist*") adj (study or studies)).mp. | 1735980 |
| 47 | ((longitudinal* or retrospective* or prospective* or observ* or cohort or case control or cross sectional or follow up or pragmatic or claims or routine care) adj stud*).mp. | 3788709 |
| 48 | Meta Analysis/ or ((meta adj analys*) or metaanalys*).ti,ab. or "systematic review"/ or ((systematic adj review) or "SLR").mp. | 710005 |
| 49 | (("real world" or "real-world") adj (data or stud* or effect* or evidence)).mp. | 37089 |
| 50 | controlled clinical trial/ or randomized controlled trial/ or controlled study/ or clinical trial/ | 10629335 |
| 51 | ((randomi?ed or control*) adj5 (trial or stud*)).mp. | 10780166 |
| 52 | or/45-51 | 13735737 |
| 53 | 44 and 52 | 5037 |

## **Table 2 Medline Search Strategy**

Database(s): **Ovid MEDLINE(R) and Epub Ahead of Print, In-Process, In-Data-Review & Other Non-Indexed Citations and Daily**1946 to June 19^th^, 2023
Search Strategy: June 21^st^, 2023

| # | Searches | Results |
| --- | --- | --- |
| 1 | *Ischemic Stroke/ | 9041 |
| 2 | (isch?emic adj3 stroke).tw. | 78130 |
| 3 | or/1-2 | 78679 |
| 4 | *hospitalization/ or *hospital patient/ or *emergency ward/ or *"length of stay"/ | 103287 |
| 5 | *morbidity/ or *mortality/ | 30375 |
| 6 | (morbidity or mortality).ti,ab. | 1140735 |
| 7 | *social care/ or *home care/ or *long term care/ or *nursing home/ or *elderly care/ | 70177 |
| 8 | ((social or home or elderly or follow*) adj3 care).ti,ab. | 79124 |
| 9 | (hospitali?ation or outpatient or inpatient or (length adj5 stay) or (emergency adj3 (ward or department))).ti,ab. | 579460 |
| 10 | (clinical adj5 (burden or impact)).ti,ab. | 54079 |
| 11 | or/4-10 | 1801600 |
| 12 | *incidence/ or *prevalence/ | 1470 |
| 13 | (incidence or prevalence or "modified rankin scale").ti,ab. | 1622710 |
| 14 | 12 or 13 | 1623070 |
| 15 | *"quality of life"/ or *quality adjusted life year/ or *disability-adjusted life year/ or *"European Quality of Life 5 Dimensions questionnaire"/ | 114567 |
| 16 | (eq adj3 5d).mp. or ("STROKE-QoL" or "SS-QoL" or "QLASS").ti,ab. | 12490 |
| 17 | (quality adj5 life).ti,ab. | 381805 |
| 18 | or/15-17 | 403290 |
| 19 | (*Health Care Economics/ and Organizations/) or *"cost of illness"/ or *"hospital cost"/ or *"hospitalization cost"/ or *"cost control"/ or *"drug cost"/ or *"health care cost"/ or *health care utilization/ or *productivity/ or medical leave/ | 83415 |
| 20 | *economic evaluation/ or *"cost benefit analysis"/ or *"cost effectiveness analysis"/ or *"cost utility analysis"/ or *cost minimization analysis/ | 17691 |
| 21 | (Cost* or expen* or financ* or price* or pricing or ((economic or societ* or socioeconomic or socio economic or illness or disease or patient* or caregiver* or carer*) adj2 burden)).ti,ab. | 1085868 |
| 22 | ((resource* adj2 (utili?ation or use*)) or productivity or hospitali?ation* or (leave adj2 (medical or sick or disability))).ti,ab. | 334416 |
| 23 | (cost adj (effective* or utilit* or minimi* or benefit)).ti,ab. | 184529 |
| 24 | or/19-23 | 1395232 |
| 25 | *practice guideline/ | 0 |
| 26 | *"health services needs and demand"/ | 23329 |
| 27 | ((clinical or treatment or therap*) adj5 (practice* or pattern* or trend* or guideline* or recommend* or barrier* or challenge*)).tw. | 601064 |
| 28 | ((unmet or me?t*) adj3 (need? or demand? or require*)).tw. | 199466 |
| 29 | or/25-28 | 809862 |
| 30 | 3 and (11 or 14 or 18 or 24 or 29) | 32939 |
| 31 | United States/ or Japan/ or France/ or Germany/ or Italy/ or Spain/ or United Kingdom/ or China/ or Wales/ or Scotland/ or England/ or Northern Ireland/ or Australia/ or Sweden/ or Denmark/ or Canada/ or Republic of Korea/ or Norway/ or Taiwan/ | 2558959 |
| 32 | (US or USA or (United adj States) or Japan or France or Germany or Italy or Spain or China or UK or (United adj Kingdom) or England or Wales or Scotland or Northern Ireland or Australia or Sweden or Denmark or Canada or South Korea or Norway or Taiwan).ti,ab. | 2198200 |
| 33 | or/31-32 | 3839764 |
| 34 | 30 and 33 | 8395 |
| 35 | limit 34 to yr="2015 -Current" | 5512 |
| 36 | exp case study/ or case report/ or letter/ or editorial/ or note/ | 3989611 |
| 37 | (editorial or letter or case report or comment or news).pt. | 2369786 |
| 38 | 35 not (36 or 37) | 5448 |
| 39 | (exp animal/ or exp invertebrate/ or nonhuman/ or animal experiment/ or animal tissue/ or animal model/ or exp plant/ or exp fungus/) not (exp human/ or human tissue/) | 5560566 |
| 40 | 38 not 39 | 5422 |
| 41 | Clinical Study/ or Case-Control Studies/ or Longitudinal Studies/ or Prospective Studies/ or Cohort Studies/ or Observational Study/ or Cross-Sectional Studies/ or Registries/ | 1902692 |
| 42 | ((Cohort or "case control" or "follow up" or observational or "cross sectional" or "regist*") adj (study or studies)).mp. | 2180420 |
| 43 | ((longitudinal* or retrospective* or prospective* or observ* or cohort or case control or cross sectional or follow up or pragmatic or claims or routine care) adj stud*).mp. | 3556142 |
| 44 | Meta Analysis/ or ((meta adj analys*) or metaanalys*).ti,ab. or "systematic review"/ or ((systematic adj review) or "SLR").mp. | 444598 |
| 45 | (("real world" or "real-world") adj (data or stud* or effect* or evidence)).mp. | 18020 |
| 46 | controlled clinical trial/ or randomized controlled trial/ or controlled study/ or clinical trial/ | 941436 |
| 47 | ((randomi?ed or control*) adj5 (trial or stud*)).mp. | 1443279 |
| 48 | or/41-47 | 4919955 |
| 49 | 40 and 48 | 3883 |

**Table 3 EconLit Search Strategy**

Database: **Econlit**1886 to June 15^th^, 2023
Search Strategy: June 21^st^, 2023

| # | Searches | Results |
| --- | --- | --- |
| 1 | acute ischemic stroke.mp. | 3 |
| 2 | (isch?emic adj3 stroke).mp. | 28 |
| 3 | or/1-2 | 28 |
| 4 | limit 3 to yr="2015 -Current" | 15 |
| 5 | (US or USA or (United adj States) or Japan or France or Germany or Italy or Spain or China or UK or (United adj Kingdom) or England or Wales or Scotland or Northern Ireland or Australia or Sweden or Denmark or Canada or South Korea or Norway or Taiwan).ti,ab. | 336253 |
| 6 | 4 and 5 | 8 |

## **Table 4 PsychINFO Search Strategy**

Database: **APA PsycInfo**1806 to June Week 2 2023
Search Strategy: June 21^st^, 2023

| # | Searches | Results |
| --- | --- | --- |
| 1 | acute ischemic stroke.mp. | 1708 |
| 2 | (isch?emic adj3 stroke).mp. | 8087 |
| 3 | or/1-2 | 8087 |
| 4 | limit 3 to yr="2015 -Current" | 3841 |
| 5 | (US or USA or (United adj States) or Japan or France or Germany or Italy or Spain or China or UK or (United adj Kingdom) or England or Wales or Scotland or Northern Ireland or Australia or Sweden or Denmark or Canada or South Korea or Norway or Taiwan).ti,ab. | 591947 |
| 6 | 4 and 5 | 423 |

## **Table 5 EBMR Search Strategy**

Database(s): **EBM Reviews - Cochrane Database of Systematic Reviews**2005 to June 14^th^, 2023**, EBM Reviews - ACP Journal Club**1991 to May 2023**, EBM Reviews - Database of Abstracts of Reviews of Effects**1^st^ Quarter 2016**, EBM Reviews - Cochrane Clinical Answers**May 2023**, EBM Reviews - Cochrane Central Register of Controlled Trials**May 2023**, EBM Reviews - Cochrane Methodology Register**3^rd^ Quarter 2012**, EBM Reviews - Health Technology Assessment**4^th^ Quarter 2016**, EBM Reviews - NHS Economic Evaluation Database**1^st^ Quarter 2016
Search Strategy: June 21^st^, 2023

| # | Searches | Results |
| --- | --- | --- |
| 1 | acute ischemic stroke.mp. | 5173 |
| 2 | (isch?emic adj3 stroke).mp. | 15664 |
| 3 | or/1-2 | 15664 |
| 4 | limit 3 to yr="2015 -Current" [Limit not valid in DARE; records were retained] | 9347 |
| 5 | (US or USA or (United adj States) or Japan or France or Germany or Italy or Spain or China or UK or (United adj Kingdom) or England or Wales or Scotland or Northern Ireland or Australia or Sweden or Denmark or Canada or South Korea or Norway or Taiwan).ti,ab. | 177209 |
| 6 | 4 and 5 | 1328 |

## **Table 6 Embase Supplementary Country Search**

Database: **Embase**1974 to 2023 June 19^th^
Search Strategy: January 4^th^, 2024.

| # | Searches | Results |
| --- | --- | --- |
| 1 | *acute ischemic stroke/ | 6,918 |
| 2 | (isch?emic adj3 stroke).tw. | 137,606 |
| 3 | or/1-2 | 137,847 |
| 4 | *hospitalization/ or *hospital patient/ or *emergency ward/ or *"length of stay"/ | 142,988 |
| 5 | *morbidity/ or *mortality/ | 137,908 |
| 6 | (morbidity or mortality).ti,ab. | 1,748,695 |
| 7 | *social care/ or *home care/ or *long term care/ or *nursing home/ or *elderly care/ | 112,785 |
| 8 | ((social or home or elderly or follow*) adj3 care).ti,ab. | 111,324 |
| 9 | (hospitali?ation or outpatient or inpatient or (length adj5 stay) or (emergency adj3 (ward or department))).ti,ab. | 1,011,776 |
| 10 | (clinical adj5 (burden or impact)).ti,ab. | 95,330 |
| 11 | or/4-10 | 2,817,642 |
| 12 | *incidence/ or *prevalence/ or rankin scale/ | 170,671 |
| 13 | (incidence or prevalence or "modified rankin scale").ti,ab. | 2,414,495 |
| 14 | 12 or 13 | 2,430,928 |
| 15 | *"quality of life"/ or *quality adjusted life year/ or *disability-adjusted life year/ or *"European Quality of Life 5 Dimensions questionnaire"/ | 144,114 |
| 16 | (eq adj3 5d).mp. or ("STROKE-QoL" or "SS-QoL" or "QLASS").ti,ab. | 24,565 |
| 17 | (quality adj5 life).ti,ab. | 628,803 |
| 18 | or/15-17 | 646,982 |
| 19 | (*Health Care Economics/ and Organizations/) or *"cost of illness"/ or *"hospital cost"/ or *"hospitalization cost"/ or *"cost control"/ or *"drug cost"/ or *"health care cost"/ or *health care utilization/ or *productivity/ or medical leave/ | 121,693 |
| 20 | *economic evaluation/ or *"cost benefit analysis"/ or *"cost effectiveness analysis"/ or *"cost utility analysis"/ or *cost minimization analysis/ | 62,442 |
| 21 | (Cost* or expen* or financ* or price* or pricing or ((economic or societ* or socioeconomic or socio economic or illness or disease or patient* or caregiver* or carer*) adj2 burden)).ti,ab. | 1,511,353 |
| 22 | ((resource* adj2 (utili?ation or use*)) or productivity or hospitali?ation* or (leave adj2 (medical or sick or disability))).ti,ab. | 539,649 |
| 23 | (cost adj (effective* or utilit* or minimi* or benefit)).ti,ab. | 261,557 |
| 24 | or/19-23 | 1,977,300 |
| 25 | *clinical practice/ | 54,859 |
| 26 | exp *practice guideline/ | 127,826 |
| 27 | *unmet medical need/ | 710 |
| 28 | ((clinical or treatment or therap*) adj5 (practice* or pattern* or trend* or guideline* or recommend* or barrier* or challenge*)).tw. | 929,062 |
| 29 | ((unmet or me?t*) adj3 (need? or demand? or require*)).tw. | 290,181 |
| 30 | or/25-29 | 1,320,026 |
| 31 | 3 and (11 or 14 or 18 or 24 or 30) | 66,205 |
| 32 | exp case study/ or case report/ or letter/ or editorial/ or note/ | 5,597,486 |
| 33 | (editorial or letter or case report or comment or news).pt. | 2,093,393 |
| 34 | 31 not (32 or 33) | 62,587 |
| 35 | (exp animal/ or exp invertebrate/ or nonhuman/ or animal experiment/ or animal tissue/ or animal model/ or exp plant/ or exp fungus/) not (exp human/ or human tissue/) | 7,815,111 |
| 36 | 34 not 35 | 60,944 |
| 37 | Clinical Study/ or Case-Control Studies/ or Longitudinal Studies/ or Prospective Studies/ or Cohort Studies/ or Observational Study/ or Cross-Sectional Studies/ or Register/ | 2,686,156 |
| 38 | ((Cohort or "case control" or "follow up" or observational or "cross sectional" or "regist*") adj (study or studies)).mp. | 1,805,501 |
| 39 | ((longitudinal* or retrospective* or prospective* or observ* or cohort or case control or cross sectional or follow up or pragmatic or claims or routine care) adj stud*).mp. | 3,920,150 |
| 40 | Meta Analysis/ or ((meta adj analys*) or metaanalys*).ti,ab. or "systematic review"/ or ((systematic adj review) or "SLR").mp. | 724,459 |
| 41 | (("real world" or "real-world") adj (data or stud* or effect* or evidence)).mp. | 40,897 |
| 42 | controlled clinical trial/ or randomized controlled trial/ or controlled study/ or clinical trial/ | 10,705,384 |
| 43 | ((randomi?ed or control*) adj5 (trial or stud*)).mp. | 10,868,641 |
| 44 | or/37-43 | 13,925,024 |
| 45 | 36 and 44 | 45,129 |
| 46 | limit 45 to yr="2015 -Current" | 33,636 |
| 47 | exp conference paper/ or conference abstract/ or (conference adj (abstract or paper or review or proceeding)).pt. | 5,820,754 |
| 48 | limit 47 to yr="2015 - 2019" | 1,987,104 |
| 49 | 46 not 48 | 25,696 |
| 50 | United States/ or Japan/ or France/ or Germany/ or Italy/ or Spain/ or United Kingdom/ or China/ or Wales/ or Scotland/ or England/ or Northern Ireland/ or Australia/ or Sweden/ or Denmark/ or Canada/ or South Korea/ or Norway/ or Taiwan/ or Finland/ or Iceland/ or Brazil/ or Mexico/ or India/ or Turkey/ or Argentina/ or Russia/ or Saudi Arabia/ or South Africa/ or Israel/ or Indonesia/ | 3,786,526 |
| 51 | (US or USA or (United adj States) or Japan or France or Germany or Italy or Spain or China or UK or (United adj Kingdom) or England or Wales or Scotland or Northern Ireland or Australia or Sweden or Denmark or Canada or Korea or Norway or Taiwan or Finland or Iceland or Brazil or Mexico or India or Turkey or Argentina or Russia or Saudi Arabia or South Africa or Israel or Indonesia).ti,ab. | 3,804,817 |
| 52 | or/50-51 | 5,666,457 |
| 53 | 49 and 52 | 6,055 |

## **Table 7 Medline Supplementary Country Search**

Database(s): **Ovid MEDLINE(R) and Epub Ahead of Print, In-Process, In-Data-Review & Other Non-Indexed Citations and Daily**1946 to June 19^th^, 2023
Search Strategy: January 4^th^, 2024.

| # | Searches | Results |
| --- | --- | --- |
| 1 | *Ischemic Stroke/ | 10,794 |
| 2 | (isch?emic adj3 stroke).tw. | 82,055 |
| 3 | or/1-2 | 82,670 |
| 4 | *hospitalization/ or *hospital patient/ or *emergency ward/ or *"length of stay"/ | 104,084 |
| 5 | *morbidity/ or *mortality/ | 30,469 |
| 6 | (morbidity or mortality).ti,ab. | 1,183,536 |
| 7 | *social care/ or *home care/ or *long term care/ or *nursing home/ or *elderly care/ | 70,718 |
| 8 | ((social or home or elderly or follow*) adj3 care).ti,ab. | 82,100 |
| 9 | (hospitali?ation or outpatient or inpatient or (length adj5 stay) or (emergency adj3 (ward or department))).ti,ab. | 604,919 |
| 10 | (clinical adj5 (burden or impact)).ti,ab. | 57,138 |
| 11 | or/4-10 | 1,868,687 |
| 12 | *incidence/ or *prevalence/ | 1,481 |
| 13 | (incidence or prevalence or "modified rankin scale").ti,ab. | 1,681,089 |
| 14 | 12 or 13 | 1,681,449 |
| 15 | *"quality of life"/ or *quality adjusted life year/ or *disability-adjusted life year/ or *"European Quality of Life 5 Dimensions questionnaire"/ | 117,210 |
| 16 | (eq adj3 5d).mp. or ("STROKE-QoL" or "SS-QoL" or "QLASS").ti,ab. | 13,296 |
| 17 | (quality adj5 life).ti,ab. | 401,476 |
| 18 | or/15-17 | 423,264 |
| 19 | (*Health Care Economics/ and Organizations/) or *"cost of illness"/ or *"hospital cost"/ or *"hospitalization cost"/ or *"cost control"/ or *"drug cost"/ or *"health care cost"/ or *health care utilization/ or *productivity/ or medical leave/ | 83,751 |
| 20 | *economic evaluation/ or *"cost benefit analysis"/ or *"cost effectiveness analysis"/ or *"cost utility analysis"/ or *cost minimization analysis/ | 17,808 |
| 21 | (Cost* or expen* or financ* or price* or pricing or ((economic or societ* or socioeconomic or socio economic or illness or disease or patient* or caregiver* or carer*) adj2 burden)).ti,ab. | 1,132,782 |
| 22 | ((resource* adj2 (utili?ation or use*)) or productivity or hospitali?ation* or (leave adj2 (medical or sick or disability))).ti,ab. | 350,966 |
| 23 | (cost adj (effective* or utilit* or minimi* or benefit)).ti,ab. | 193,170 |
| 24 | or/19-23 | 1,455,854 |
| 25 | *practice guideline/ | 0 |
| 26 | *"health services needs and demand"/ | 23,369 |
| 27 | ((clinical or treatment or therap*) adj5 (practice* or pattern* or trend* or guideline* or recommend* or barrier* or challenge*)).tw. | 629,436 |
| 28 | ((unmet or me?t*) adj3 (need? or demand? or require*)).tw. | 209,644 |
| 29 | or/25-28 | 847,714 |
| 30 | 3 and (11 or 14 or 18 or 24 or 29) | 34,867 |
| 31 | United States/ or Japan/ or France/ or Germany/ or Italy/ or Spain/ or United Kingdom/ or China/ or Wales/ or Scotland/ or England/ or Northern Ireland/ or Australia/ or Sweden/ or Denmark/ or Canada/ or Republic of Korea/ or Norway/ or Taiwan/ or Finland/ or Iceland/ or Brazil/ or Mexico/ or India/ or Turkey/ or Argentina/ or Russia/ or Saudi Arabia/ or South Africa/ or Israel/ or Indonesia/ | 3,096,683 |
| 32 | (US or USA or (United adj States) or Japan or France or Germany or Italy or Spain or China or UK or (United adj Kingdom) or England or Wales or Scotland or Northern Ireland or Australia or Sweden or Denmark or Canada or Korea or Norway or Taiwan or Finland or Iceland or Brazil or Mexico or India or Turkey or Argentina or Russia or Saudi Arabia or South Africa or Israel or Indonesia).ti,ab. | 2,770,244 |
| 33 | or/31-32 | 4,623,609 |
| 34 | 30 and 33 | 9,727 |
| 35 | limit 34 to yr="2015 -Current" | 6,494 |
| 36 | exp case study/ or case report/ or letter/ or editorial/ or note/ | 4,068,673 |
| 37 | (editorial or letter or case report or comment or news).pt. | 2,423,091 |
| 38 | 35 not (36 or 37) | 6,420 |
| 39 | (exp animal/ or exp invertebrate/ or nonhuman/ or animal experiment/ or animal tissue/ or animal model/ or exp plant/ or exp fungus/) not (exp human/ or human tissue/) | 5,617,641 |
| 40 | 38 not 39 | 6,391 |
| 41 | Clinical Study/ or Case-Control Studies/ or Longitudinal Studies/ or Prospective Studies/ or Cohort Studies/ or Observational Study/ or Cross-Sectional Studies/ or Registries/ | 1,951,138 |
| 42 | ((Cohort or "case control" or "follow up" or observational or "cross sectional" or "regist*") adj (study or studies)).mp. | 2,245,656 |
| 43 | ((longitudinal* or retrospective* or prospective* or observ* or cohort or case control or cross sectional or follow up or pragmatic or claims or routine care) adj stud*).mp. | 3,669,633 |
| 44 | Meta Analysis/ or ((meta adj analys*) or metaanalys*).ti,ab. or "systematic review"/ or ((systematic adj review) or "SLR").mp. | 474,532 |
| 45 | (("real world" or "real-world") adj (data or stud* or effect* or evidence)).mp. | 20,868 |
| 46 | controlled clinical trial/ or randomized controlled trial/ or controlled study/ or clinical trial/ | 953,631 |
| 47 | ((randomi?ed or control*) adj5 (trial or stud*)).mp. | 1,476,483 |
| 48 | or/41-47 | 5,080,729 |
| 49 | 40 and 48 | 4,533 |

## **Table 8 EconLit Supplementary Country Search**

Database: **Econlit**1886 to June 19^th^, 2023
Search Strategy: January 4^th^, 2024.

| **#** | **Searches** | **Results** |
| --- | --- | --- |
| 1 | acute ischemic stroke.mp. | 3 |
| 2 | (isch?emic adj3 stroke).mp. | 29 |
| 3 | or/1-2 | 29 |
| 4 | limit 3 to yr="2015 -current" | 16 |
| 5 | (US or USA or (United adj States) or Japan or France or Germany or Italy or Spain or China or UK or (United adj Kingdom) or England or Wales or Scotland or Northern Ireland or Australia or Sweden or Denmark or Canada or Korea or Norway or Taiwan or Finland or Iceland or Brazil or Mexico or India or Turkey or Argentina or Russia or Saudi Arabia or South Africa or Israel or Indonesia).ti,ab. | 426620 |
| 6 | 4 and 5 | 9 |

## **Table 9 PsychINFO Supplementary Country Search**

Database: **APA PsycInfo**1806 to June Week 3 2024
Search Strategy:

| **#** | **Searches** | **Results** |
| --- | --- | --- |
| 1 | acute ischemic stroke.mp. | 1783 |
| 2 | (isch?emic adj3 stroke).mp. | 8397 |
| 3 | or/1-2 | 8397 |
| 4 | limit 3 to yr="2015 - current" | 4095 |
| 5 | (US or USA or (United adj States) or Japan or France or Germany or Italy or Spain or China or UK or (United adj Kingdom) or England or Wales or Scotland or Northern Ireland or Australia or Sweden or Denmark or Canada or Korea or Norway or Taiwan or Finland or Iceland or Brazil or Mexico or India or Turkey or Argentina or Russia or Saudi Arabia or South Africa or Israel or Indonesia).ti,ab. | 704504 |
| 6 | 4 and 5 | 504 |

## **Table 10 EBMR Supplementary Country Search**

Database(s): **EBM Reviews - Cochrane Database of Systematic Reviews**2005 to March 20^th^, 2024**, EBM Reviews - ACP Journal Club**1991 to June, 2023**, EBM Reviews - Database of Abstracts of Reviews of Effects**1^st^ Quarter 2016**, EBM Reviews - Cochrane Clinical Answers**March 2024**, EBM Reviews - Cochrane Central Register of Controlled Trials**February 2024**, EBM Reviews - Cochrane Methodology Register**3^rd^ Quarter 2012**, EBM Reviews - Health Technology Assessment**4^th^ Quarter 2016**, EBM Reviews - NHS Economic Evaluation Database**1^st^ Quarter 2016
Search Strategy: January 4^th^, 2024.

| **#** | **Searches** | **Results** |
| --- | --- | --- |
| 1 | acute ischemic stroke.mp. | 5596 |
| 2 | (isch?emic adj3 stroke).mp. | 16775 |
| 3 | or/1-2 | 16775 |
| 4 | limit 3 to yr="2015 - current" [Limit not valid in DARE; records were retained] | 10328 |
| 5 | (US or USA or (United adj States) or Japan or France or Germany or Italy or Spain or China or UK or (United adj Kingdom) or England or Wales or Scotland or Northern Ireland or Australia or Sweden or Denmark or Canada or Korea or Norway or Taiwan or Finland or Iceland or Brazil or Mexico or India or Turkey or Argentina or Russia or Saudi Arabia or South Africa or Israel or Indonesia).ti,ab. | 214106 |
| 6 | 4 and 5 | 1591 |

**Table 11 PICOTs Eligibility Criteria**

|  | **Inclusion criteria** | **Exclusion criteria** |
| --- | --- | --- |
| **Population** | 1. Patients with AIS 2. For clinical burden outcomes only patients with moderate to severe AIS reported as any of the following:  - Patients reported as ‘moderate’ to ‘severe.’ - Median NIHSS score ≥8 - At least 80% of patients with a NIHSS ≥5^*^ (where no stratified data is reported).  1. Studies on the following comorbid conditions as they relate to AIS outcomes (and risk of AIS) were also included:  - Atrial fibrillation - Heart disease - Metabolic conditions (e.g., diabetes, obesity, high blood pressure, hyperlipidemia etc.) | 1. Mixed populations which do not report segregated AIS results (e.g., includes hemorrhagic stroke cases). 2. Studies which do not explicitly include patients with AIS in the abstract. 3. Children and adolescents. 4. Studies <500 patients (clinical burden only). |
| **Intervention/ Comparator** | 1. N/A | 1. Studies assessing the impact of treatment on disease outcomes in AIS patients (e.g., comparing different treatment options; excluding economic burden and economic evaluations). 2. Traditional Chinese medicines 3. Lifestyle interventions (e.g., dietary, exercise etc.) |
| **Outcomes** | 1. Economic Burden  - Direct costs - Indirect costs - Resource use  1. Post-stroke HCRU 2. Number of days in hospital 3. Time in healthcare institution and type of healthcare institution 4. Number of office visits  - Non-medical costs  1. Economic evaluations  - QALYs - ICERs - Total costs - Life years gained. - Utility outcomes  1. Clinical burden  - Incidence - Prevalence - Mortality (stratified by all-cause and stroke-related mortality where reported) - Morbidity - Severity of stroke (e.g., NIHSS score) - Hospitalization rates - Discharge status and level of care received. - Life expectancy - Impairment (e.g., mRS) - Reperfusion achieved:   - TICI scale  1. Humanistic burden  - PROs (e.g., any measure of HRQoL) - Caregiver burden - Measures of physical function  1. Treatment guidelines  - Publications reporting real-world treatment patterns and guidelines.  1. Other measures of burden:  - Unmet need | 1. N/A |
| **Study design** | 1. Observational studies (e.g., cohort, case-control, cross-sectional) 2. Studies reporting economic burden and cost-effectiveness data. 3. Treatment guidelines 4. Practice patterns | 1. Randomized controlled trials** 2. Single arm studies 3. Case reports / case series 4. Letters 5. Editorials 6. Notes 7. Expert opinions / commentaries 8. Erratum 9. News articles 10. Studies with no results (e.g., protocol, methodology) 11. Animal studies 12. Literature reviews |
| **Geographical limits** | 1. Argentina, Australia, Brazil, Canada, China, Denmark, EU4 (France, Germany, Italy, and Spain), Finland, Iceland, Israel, India, Indonesia, Japan, Mexico, Norway, Russia, Sweden, Saudi Arabia, South Africa, South Korea, Taiwan, Turkey, UK, and US | 1. N/A |

*80% was chosen as the threshold as this implies that the majority of the population could be considered ‘moderate’ or ‘severe’.

**This SLR excluded RCT studies as they are conducted in a controlled environment and may not reflect care provided in clinical practice and thus not reflective of burden of disease or cost of care.

AIS: Acute ischemic stroke; EU4: European Union 4; HCRU: Healthcare resource utilization; HRQoL: Health-related quality of life; ICER: Incremental cost-effectiveness ratio; mRS: Modified Ranking Scale; N/A: Not applicable;
NIHSS: National Institutes of Health Stroke Scale; POR: Patient reported outcomes; QALY: Quality-adjusted life years; TICI: Thrombolysis in cerebral infarction; UK: United Kingdom; US: United States.

**Table 12 Clinical burden studies reporting mortality data**

| **Author, year** | **Study population** | **N** | **Baseline mean age (SD)** | **Female (%)** | **Baseline median (IQR) NIHSS** | **Data collection period** | **Time point** | **Outcome description** | **Statistical measure** | **Estimate** | **Unit** |
| --- | --- | --- | --- | --- | --- | --- | --- | --- | --- | --- | --- |
| **China** | | | | | | | | | | | |
| Ma, 2022(1) | Patients with AIS (with DPT) | 932 | 65.1 | 39.9 | 16 (13–20) | Nov 2017 – Mar 2019 | 3 months | All-cause mortality | Total | 16.40 | % |
|  | Patients with AIS (with PRT) | 815 | 64.9 | 39.9 | 16 (12–20) | Nov 2017 – Mar 2019 | 3 months | All-cause mortality | Total | 15.10 | % |
| Gao, 2021(2) | Patients with AIS after EVT (1st tertile of serum albumin levels) | 203 | 64.2 (12.4) | 40.7 | 18 (13–23) | Jan 2014 – Jun 2016 | 3 months | All-cause mortality | Total | 68 (33.5) | N (%) |
|  | Patients with AIS after EVT (2nd tertile of serum albumin levels) | 202 | 64.2 (12.4) | 40.7 | 16 (12–20) | Jan 2014 – Jun 2016 | 3 months | All-cause mortality | Total | 58 (28.7) | N (%) |
|  | Patients with AIS after EVT (3rd tertile of serum albumin levels) | 200 | 64.2 (12.4) | 40.7 | 15 (12–19) | Jan 2014 – Jun 2016 | 3 months | All-cause mortality | Total | 29 (14.5) | N (%) |
| Wu, 2021(3) | Patients with moderate LAA AIS | 480 | 70.6 | 38.3 | 8–16 [range] | Jan 2016 – Dec 2018 | 3 months | All-cause mortality | Total | 45 (10.2) | N (%) |
|  | Patients with moderate LAA AIS | 480 | 70.6 | 38.3 | 8–16 [range] | Jan 2016 – Dec 2018 | 12 months | All-cause mortality | Total | 53 (16.6) | N (%) |
|  | Patients with moderate LAA AIS | 480 | 70.6 | 38.3 | 8–16 [range] | Jan 2016 – Dec 2018 | 36 months | All-cause mortality | Total | 39 (36.8) | N (%) |
|  | Patients with severe LAA AIS | 172 | 70.6 | 38.3 | ≥17 [range] | Jan 2016 – Dec 2018 | 3 months | All-cause mortality | Total | 41 (29.9) | N (%) |
|  | Patients with severe LAA AIS | 172 | 70.6 | 38.3 | ≥17 [range] | Jan 2016 – Dec 2018 | 12 months | All-cause mortality | Total | 47 (49.0) | N (%) |
|  | Patients with severe LAA AIS | 172 | 70.6 | 38.3 | ≥17 [range] | Jan 2016 – Dec 2018 | 36 months | All-cause mortality | Total | 23 (67.6) | N (%) |
| Yang, 2021(4) | Patients with AIS (neurologist-dominant model) | 543 | 66 [median] | 40.7 | 16 (12–21) | Jan 2014 – Jun 2016 | 90 days | All-cause mortality | Total | 145 (26.7) | N (%) |
|  | Patients with AIS (non-neurologist-dominant model) | 89 | 64 [median] | 48.3 | 17 (12–21) | Jan 2014 – Jun 2016 | 90 days | All-cause mortality | Total | 21 (23.6) | N (%) |
|  | Patients with AIS (neurologist-dominant model) | 543 | 66 [median] | 40.7 | 16 (12–21) | Jan 2014 – Jun 2016 | 90 days | All-cause mortality | Total | 22 (25.0) | N (%) |
|  | Patients with AIS (non-neurologist-dominant model) | 89 | 64 [median] | 48.3 | 17 (12–21) | Jan 2014 – Jun 2016 | 90 days | All-cause mortality | Total | 20 (22.7) | N (%) |
| Wang, 2017(5) | Patients with AIS | 873 | 40–75 [range] | NR | >10 [range] | Jan 2013 – June 2014 | 3 months | All-cause mortality | Total | 33.00 | N |
|  | Patients with AIS | 873 | 40–75 [range] | NR | >10 [range] | Jan 2013 – June 2014 | 12 months | All-cause mortality | Total | 83.00 | N |
| **Italy** | | | | | | | | | | | |
| Scrutino, 2020(6) | Patients with severe AIS as defined by the Medicare case-mix classification system | 1,316 | 72 | 44.6 | NR | Feb 2002 – Sept 2016 | 36 months | All-cause mortality | Crude HR | 0.94 (0.74–1.20) | NR (95% CI) |
|  | Patients with severe AIS as defined by the Medicare case-mix classification system | 1,316 | 72 | 44.6 | NR | Feb 2002 – Sept 2016 | 36 months | All-cause mortality | Adjusted HR | 0.73 (0.56–0.96) | NR (95% CI) |
|  | Patients with severe AIS as defined by the Medicare case-mix classification system | 1,316 | 72 | 44.6 | NR | Feb 2002 – Sept 2016 | 36 months | All-cause mortality | Fully adjusted HR | 0.73 (0.56–0.95) | NR (95% CI) |
| **Japan** | | | | | | | | | | | |
| Uchida, 2019(7) | Patients with AIS (females) | 1,087 | 79.7 | 100 | 18 (12–24) | NR | 90 days | All-cause mortality | Total | 134 (12.3) | N (%) |
|  | Patients with AIS (males) | 1,312 | 72.8 | 0 | 16 (9–22) | NR | 90 days | All-cause mortality | Total | 130 (9.9) | N (%) |
|  | Patients with AIS (females vs. males) | NR | NR | NR | NR | NR | 90 days | All-cause mortality | Crude OR | 1.28 (0.99–1.65) | NR (95% CI) |
|  | Patients with AIS (females vs. males) | NR | NR | NR | NR | NR | 90 days | All-cause mortality | Adjusted OR | 0.78 (0.58–1.05) | NR (95% CI) |
| **Mexico** | | | | | | | | | | | |
| Aguilar-Salas, 2022(8) | Adult patients with AIS | 934 | 69.2 (14.3) | 52.1 | 12 (7–20) | Apr 2018 – Sept 2019 | 18 months | All-cause mortality | Case fatality rate | 28.70 (25.8–31.6) | % (95% CI) |
| Arauz, 2018(9) | Patients with AIS | 3,509 | 55 (42–68) [median (IQR)] | NR | 10 (5–16) | Jan 1900 – Jan 2015 | NR | All-cause mortality | Total | 18.00 | % |
| **Saudi Arabia** | | | | | | | | | | | |
| Al Khathaami, 2020(10) | Patients with AIS (LVO) | 415 | 60.48 (12.49) | 35.90 | 9 (10) | Feb 2016 – Jul 2019 | NR | In-hospital mortality rate | Total | 27 (6.51) | N (%) |
|  | Patients with AIS (non-LVO) | 1,080 | 58.52 (14.00) | 36.11 | 4 (4) | Feb2016 – Jul 2019 | NR | In-hospital mortality rate | Total | 15 (1.39) | N (%) |
| **South Korea** | | | | | | | | | | | |
| Lee, 2021(11) | Patients with AIS arriving within 0–4.5 hours | 153 | 68.3 (13.1) | 35.3 | 5.55 (6.70) [mean (SD)] | Sept 2019 – May 2020 | 3 months | All-cause mortality | NR | 0 (0%) | N (%) |
| Park, 2017(12) | Patients with AIS (no IVT) | 181 | 69 (12) | 43 | 15 (10–18.5) | Apr 2008 – Jul 2013 | 3 months | All-cause mortality | Total | 44 (24) | N (%) |
|  | Patients with AIS (preceding IVT) | 458 | 69 (12) | 43 | 15 (11–19) | Apr 2008 – Jul 2013 | 3 months | All-cause mortality | Total | 68 (15) | N (%) |
| Yoon, 2017(13) | Patients with AIS (J index* <3.31 and initial glucose ≤180mg/dL) | 127 | 67.8 | 40.5 | 5.69 (5.5) [mean (SD)] | Mar 2005 – Dec 2014 | 3 months | All-cause mortality | Total | 9.40 | % |
|  | Patients with AIS (J index* <3.31 and initial glucose >180mg/dL) | 36 | 67.8 | 40.5 | 5.69 (5.5) [mean (SD)] | Mar 2005 – Dec 2014 | 3 months | All-cause mortality | Total | 8.30 | % |
|  | Patients with AIS (J index* 3.32-4.55 and initial glucose ≤180mg/dL) | 108 | 69 | 38.7 | 5.54 (5.9) [mean (SD)] | Mar 2005 – Dec 2014 | 3 months | All-cause mortality | Total | 7.40 | % |
|  | Patients with AIS (J index* 3.32-4.55 and initial glucose >180mg/dL) | 60 | 69 | 38.7 | 5.54 (5.9) [mean (SD)] | Mar 2005 – Dec 2014 | 3 months | All-cause mortality | Total | 3.30 | % |
|  | Patients with AIS (J index* 4.56-6.54 and initial glucose ≤180mg/dL) | 89 | 66.5 | 37.9 | 5.60 (5.6) [mean (SD)] | Mar 2005 – Dec 2014 | 3 months | All-cause mortality | Total | 3.40 | % |
|  | Patients with AIS (J index* 4.56-6.54 and initial glucose >180mg/dL) | 77 | 66.5 | 37.9 | 5.60 (5.6) [mean (SD)] | Mar 2005 – Dec 2014 | 3 months | All-cause mortality | Total | 11.70 | % |
|  | Patients with AIS (J index* >6.55 and initial glucose ≤180mg/dL) | 53 | 68.9 | 43.2 | 7.36 (7.3) [mean (SD)] | Mar 2005 – Dec 2014 | 3 months | All-cause mortality | Total | 15.10 | % |
|  | Patients with AIS (J index* >6.55 and initial glucose >180mg/dL) | 114 | 68.9 | 43.2 | 7.36 (7.3) [mean (SD)] | Mar 2005 – Dec 2014 | 3 months | All-cause mortality | Total | 13.20 | % |
| **Taiwan** | | | | | | | | | | | |
| Chen, 2021(14) | Young adult patients with AIS | 670 | 39.9 (4.2) | 32.4 | 8.1 (10.4) [mean (SD)] | Feb 2009 – Dec 2019 | 1 year | All-cause mortality | Total | 90 (13.6) | N (%) |
| Ong, 2017(15) | Patients with AIS | 444 | 69.89 (12.12) | 49.3 | 16–25 [range] | Jan 2007 – Dec 2014 | NR | Risk of in-hospital mortality vs. NIHSS ≤5 | OR | 7.79 (3.68–16.48) | NR (95% CI) |
|  | Patients with AIS (females) | 219 | 72.18 (12.18) | 100 | 16–25 [range] | Jan 2007 – Dec 2014 | NR | Risk of in-hospital mortality vs. NIHSS ≤5 | OR | 6.07 (2.08–17.70) | NR (95% CI) |
|  | Patients with AIS (males) | 225 | 68.29 (11.81) | 0 | 16–25 [range] | Jan 2007 – Dec 2014 | NR | Risk of in-hospital mortality vs. NIHSS ≤5 | OR | 10.10 (3.39–30.07) | NR (95% CI) |
|  | Patients with AIS | 253 | 69.89 (12.12) | 58.1 | >25 [range] | Jan 2007 – Dec 2014 | NR | Risk of in-hospital mortality vs. NIHSS ≤5 | OR | 22.24 (10.17–47.52) | NR (95% CI) |
|  | Patients with AIS (females) | 147 | 72.18 (12.18) | 100 | >25 [range] | Jan 2007 – Dec 2014 | NR | Risk of in-hospital mortality vs. NIHSS ≤5 | OR | 17.90 (6.09–52.67) | NR (95% CI) |
|  | Patients with AIS (males) | 106 | 68.29 (11.81) | 0 | >25 [range] | Jan 2007 – Dec 2014 | NR | Risk of in-hospital mortality vs. NIHSS ≤5 | OR | 24.48 (9.46–85.78) | NR (95% CI) |
| **US** | | | | | | | | | | | |
| Mueller-Kronast, 2017(16) | Patients with AIS | 984 | 67.8 | 45.8 | 17 (13–22) | August 2014 – June 2016 | 90 days | All-cause mortality | Total | 142 (14.4) | N (%) |
|  | Patients with AIS (successful reperfusion) | 724 | 67.8 | 45.8 | 17 (13–22) | August 2014 – June 2016 | 90 days | All-cause mortality | Total | 12.70 | % |

*The J-index is a measure of quality of glycemic control based on the combination of information from the mean and SD calculated as ‘0.001 x (mean + SD)’ for glucose measured in mg/dL or 0.324 x (mean + SD) for glucose measure in mmol/L.

AIS: acute ischemic stroke; CI: confidence interval; DPT: door-to-puncture time; DRT: door-to-reperfusion time; EVT: endovascular therapy; HR: hazard ratio; ICH: intracranial hemorrhage; IQR: interquartile range; IVT: intravenous thrombolysis; LAA: large-artery atherosclerosis; NIHSS: National Institutes of Health Stroke Scale; NR: not reported; OR: odds ratio; PRT: puncture-to-reperfusion time; SSS: Scandinavian Stroke Scale; TIA: transient ischemic attack

1. Ma G, Yu Z, Jia B, Xian Y, Ren Z, Mo D, et al. Time to Endovascular Reperfusion and Outcome in Acute Ischemic Stroke: A Nationwide Prospective Registry in China. Clinical Neuroradiology. 2022;32(4):997-1009.

2. Gao J, Zhao Y, Du M, Guo H, Wan T, Wu M, et al. Serum albumin levels and clinical outcomes among ischemic stroke patients treated with endovascular thrombectomy. Neuropsychiatric Disease and Treatment. 2021;17:401-11.

3. Wu Q, Cui J, Xie Y, Wang M, Zhang H, Hu X, et al. Outcomes of Ischemic Stroke and Associated Factors Among Elderly Patients With Large-Artery Atherosclerosis: A Hospital-Based Follow-Up Study in China. Frontiers in Neurology. 2021;12 (no pagination).

4. Yang D, Zi W, Wang H, Hao Y, Zhou Z, Lin M, et al. Impacts of in-hospital workflow on functional outcome in stroke patients treated with endovascular thrombectomy. Journal of Thrombosis and Thrombolysis. 2021;51(1):203-11.

5. Wang Y, Wang J, Meng P, Liu N, Ji N, Zhang G, et al. Mid-Term Blood Pressure Variability Is Associated With Clinical Outcome After Ischemic Stroke. American Journal of Hypertension. 2017;30(10):968-77.

6. Scrutinio D, Battista P, Guida P, Lanzillo B, Tortelli R. Sex Differences in Long-Term Mortality and Functional Outcome After Rehabilitation in Patients With Severe Stroke. Frontiers in Neurology. 2020;11 (no pagination).

7. Uchida K, Yoshimura S, Sakai N, Yamagami H, Morimoto T. Sex Differences in Management and Outcomes of Acute Ischemic Stroke with Large Vessel Occlusion. Stroke. 2019;50(7):1915-8.

8. Aguilar-Salas ER-A, G. Garcia-Dominguez, K. Garfias-Guzman, C. Hernandez-Camarillo, E. Oropeza-Bustos, N. Arguelles-Castro, R. Mitre-Salazar, A. Garcia-Torres, G. Reynoso-Marenco, M. Morales-Andrade, E. Gervacio-Blanco, L. Garcia-Lopez, V. Valiente-Herves, G. Martinez-Marino, M. Flores-Silva, F. Chiquete, E. Cantu-Brito, C. Acute Stroke Care in Mexico City: The Hospital Phase of a Stroke Surveillance Study. Brain Sciences. 2022;12(7) (no pagination).

9. Arauz AM-R, J. M. Barboza, M. A. Serrano, F. Artigas, C. Murillo-Bonilla, L. M. Cantu-Brito, C. Ruiz-Sandoval, J. L. Barinagarrementeria, F. Mexican-National Institute of Neurology and Neurosurgery-Stroke Registry: Results of a 25-year hospital-based study. Frontiers in Neurology. 2018;9(APR) (no pagination).

10. Al Khathaami AMA, M. A. Al Bdah, B. A. Alhasson, M. A. Alsaif, S. A. Alluhidan, W. A. Almutairi, F. M. Alskaini, M. A. Alotaibi, N. Alghamdi, S. A. M. Anterior circulation large vessel occlusion stroke in Saudi Arabia: Prevalence, predictors, and outcome. Journal of Stroke and Cerebrovascular Diseases. 2020;29(9) (no pagination).

11. Lee EJ, Kim SJ, Bae J, Lee EJ, Kwon OD, Jeong HY, et al. Impact of onset-to-door time on outcomes and factors associated with late hospital arrival in patients with acute ischemic stroke. PLoS ONE. 2021;16(3 March) (no pagination).

12. Park HKC, J. W. Hong, J. H. Jang, M. U. Noh, H. D. Park, J. M. Kang, K. Lee, S. J. Ko, Y. Kim, J. G. Cha, J. K. Kim, D. H. Nah, H. W. Han, M. K. Kim, B. J. Park, T. H. Park, S. S. Lee, K. B. Hong, K. S. Cho, Y. J. Lee, B. C. Yu, K. H. Oh, M. S. Cho, K. H. Kim, J. T. Kim, D. E. Ryu, W. S. Choi, J. C. Kim, W. J. Shin, D. I. Yeo, M. J. Sohn, S. I. Lee, J. S. Lee, J. Yoon, B. W. Bae, H. J. Preceding Intravenous Thrombolysis in Patients Receiving Endovascular Therapy. Cerebrovascular Diseases. 2017;44(1-2):51-8.

13. Yoon JE, Sunwoo JS, Kim JS, Roh H, Ahn MY, Woo HY, et al. Poststroke glycemic variability increased recurrent cardiovascular events in diabetic patients. J Diabetes Complications. 2017;31(2):390-4.

14. Chen M, Luo W, Li J, Cao K, Li X, Huang H, et al. Clinical Characteristics and Outcomes of Acute Ischemic Stroke in Patients with Type 2 Diabetes: A Single-Center, Retrospective Study in Southern China. International Journal of Endocrinology. 2021;2021 (no pagination).

15. Ong E, Barraco F, Nighoghossian N, Praire A, Desestret V, Derex L, et al. Cerebrovascular events as presenting manifestations of Myeloproliferative Neoplasm. Revue Neurologique. 2016;172(11):703-8.

16. Mueller-Kronast NH, Zaidat OO, Froehler MT, Jahan R, Aziz-Sultan MA, Klucznik RP, et al. Systematic evaluation of patients treated with neurothrombectomy devices for acute ischemic stroke primary results of the STRATIS registry. Stroke. 2017;48(10):2760-8.

**Table 13 Studies reporting on depression and cognitive impairment**

| Author, Year | Study population | N | Mean Age (SD) | Female (%) | Timepoint | Sample Size | Outcome Description | Assessment Tool | Estimate, N (%) [SD] | P-Value |
| --- | --- | --- | --- | --- | --- | --- | --- | --- | --- | --- |
| **US** | | | | | | | | | | |
| Horn, 2022 | Patients with AIS + post stroke dysphagia | 9,163 | 78.66 (NR) | 53.49 | 2017 | 1,440 | Depression | ICD-10-CM diagnosis codes | 173 (12.01) | 0.003 |
|  | Patients with AIS without dysphagia |  |  |  |  | 7,723 |  |  | 735 (9.52) |  |
|  | Patients with AIS + post stroke dysphagia |  |  |  |  | 1,440 | Cognitive impairment |  | 421 (29.24) | <0.0001 |
|  | Patients with AIS without dysphagia |  |  |  |  | 7,723 |  |  | 1,462 (18.93) |  |
| Mayman, 2021 | Patients with AIS + depression | 174,901 | >75.00 (NR) | 51.70 | 1.5-Years post-stroke | 174,901 | Cumulative risk of depression after index admission | ICD-10-CM diagnosis codes | 0.16 [0.00] | 0.0001 |
| Katzan, 2018 | Patients with AIS | 1,195 | 62.00 (NR) | 45.10 | February 2015 – January 2017 | 1,120 | PROMIS depression | PROMIS | 49.8 [10.80] | (NR) |
| **China** | | | | | | | | | | |
| She, 2021 | Male patients with AIS | 1,714 | 61.40 (NR) | 36.60 | January 2016 –February 2017 | 1,087 | Cognitive impairment | MoCA score <24 | 683 (62.80) | (NR) |
|  | Female patients with AIS |  |  |  |  | 627 |  |  | 544 (86.80) |  |
|  | Patients with AIS |  |  |  |  | 1,714 |  |  | 1,227 (71.60) | <0.001 |
| Wang, 2017 | Patients with AIS | 1,094 | 68.3 (NR) | 38.7 | 2 Weeks Post-stroke | 1,094 | Prevalence of PSD | HAMD-17 | 25.4 | (NR) |
|  |  |  |  |  | 3 Months Post-stroke |  |  |  | 17.6 |  |
|  |  |  |  |  | 12 Months Post-stroke |  |  |  | 12.4 |  |
|  | Female patients with AIS |  |  |  | 2 Weeks Post-stroke | 34.6% |  |  | 31.4 | <0.001 |
|  |  |  |  |  | 3 Months Post-stroke |  |  |  | 20.1 | 0.11 |
|  |  |  |  |  | 12 Months Post-stroke |  |  |  | 15.0 | 0.06 |
|  | Male patients with AIS |  |  |  | 2 Weeks Post-stroke | 76.4% |  |  | 22.2 | <0.001 |
|  |  |  |  |  | 3 Months Post-stroke |  |  |  | 16.2 | 0.11 |
|  |  |  |  |  | 12 Months Post-stroke |  |  |  | 11.1 | 0.06 |
| **South Korea** | | | | | | | | | | |
| Yoon, 2017 | Stable patients with AIS (Normal Cognition) | 1,735 | 60.00 (NR) | 38.00 | 3 Months Post-stroke | 1,430 (82.4%) | Cognitive impairment | K-MMSE | 28.09 [2.66] | (NR) |
|  | Converter patients with AIS (Normal Cognition) |  |  |  |  | 93 (5.4%) |  |  | 23.67 [3.60] |  |
|  | Reverted patients with AIS (Normal Cognition) |  |  |  |  | 212 (12.2%) |  |  | 26.81 [3.53] |  |
|  | Stable patients with AIS (Declined Cognition) | 890 |  |  |  | 472 (53.0%) |  |  | 16.14 [9.81] |  |
|  | Converter patients with AIS (Declined Cognition) |  |  |  |  | 321 (36.1%) |  |  | 17.87 [9.81] |  |
|  | Reverter patients with AIS (Declined Cognition) |  |  |  |  | 97 (10.9%) |  |  | 16.34 [7.27] |  |
|  | Stable patients with AIS (Normal Cognition) | 1,735 |  |  | 12 Months Post-stroke | 1,430 (82.4%) |  |  | 28.14 [2.75] |  |
|  | Converter patients with AIS (Normal Cognition) |  |  |  |  | 93 (5.4%) |  |  | 27.41 [3.19] |  |
|  | Reverter patients with AIS (Normal Cognition) |  |  |  |  | 212 (12.2%) |  |  | 22 [4.80] |  |
|  | Stable patients with AIS (Declined Cognition) | 890 |  |  |  | 472 (53.0%) |  |  | 16.42 [10.09] |  |
|  | Converter patients with AIS (Declined Cognition) |  |  |  |  | 321 (36.1%) |  |  | 24.11 [5.69] |  |
|  | Reverter patients with AIS (Declined Cognition) |  |  |  |  | 97 (10.9%) |  |  | 11.03 [7.46] |  |
|  | Stable older aged patients with AIS (Normal Cognition) | 835 |  |  | 3 Months Post-stroke | 612 (73.3%) |  |  | 26.86 [3.41] |  |
|  | Converter older aged patients with AIS (Normal Cognition) |  |  |  |  | 79 (9.5%) |  |  | 23.18 [3.69] |  |
|  | Reverter older aged patients with AIS (Normal Cognition) |  |  |  |  | 144 (17.2%) |  |  | 25.85 [3.82] |  |
|  | Stable older aged patients with AIS (Declined Cognition) | 596 |  |  |  | 336 (56.4%) |  |  | 14.46 [9.71] |  |
|  | Converter older aged patients with AIS (Declined Cognition) |  |  |  |  | 183 (30.7%) |  |  | 15.95 [7.64] |  |
|  | Reverter older aged patients with AIS (Declined Cognition) |  |  |  |  | 77 (12.95) |  |  | 16.08 [7.09] |  |
|  | Stable older aged patients with AIS (Normal Cognition) | 835 |  |  | 12 Months Post-stroke | 612 (73.3%) |  |  | 26.88 [3.50] |  |
|  | Converter older age patients with AIS (Normal Cognition) |  |  |  |  | 79 (9.5%) |  |  | 27.01 [3.30] |  |
|  | Reverter older aged patients with AIS (Normal Cognition) |  |  |  |  | 144 (17.2%) |  |  | 20.66 [4.90] |  |
|  | Stable older aged patients with AIS (Declined Cognition) | 596 |  |  |  | 336 (56.4%) |  |  | 14.68 [9.95] |  |
|  | Converter older aged patients with AIS (Declined Cognition) |  |  |  |  | 183 (30.7%) |  |  | 22.46 [5.78] |  |
|  | Reverter older aged patients with AIS (Declined Cognition) |  |  |  |  | 77 (12.95) |  |  | 10.52 [7.21] |  |

AIS: acute ischemic stroke; ASU: acute stroke unit; 95% CI: 95% confidence interval; ICD-10: international classification of diseases; K-MMSE: Korean mini mental state examination; MoCA: Montreal cognitive assessment; PRO: patient-reported outcome; PSD: post-stroke depression; PROMIS: patient-reported outcomes measurement information system; QoL: quality-of-life; SD: standard deviation; US: United States

Table 14 Economic burden studies - costs

| Author, Year | Study Population | Data source | Mean age (SD) | Female (%) | Timepoint | Sample size | Cost Description | Statistical measure | Currency (Year) | Estimate | P-Value |
| --- | --- | --- | --- | --- | --- | --- | --- | --- | --- | --- | --- |
| **US** | | | | | | | | | | | |
| Kwok, 2023(1) | MT patients treated with IA thrombolytics | National Inpatient Sample | >65 years = 61.8% | 51.8% | 2017 –  2019 | 1,990 | Inpatient costs | Median | USD (NR) | 36,992.00 | 0.370 |
|  | MT patients treated without IA thrombolytics |  | >65 years = 61.8% | 51.8% | 2017 –  2019 | 1,990 | Inpatient costs | Median | USD (NR) | 35,440.00 | 0.370 |
| Qureshi, 2022(2) | Patients with AIS + dysphagia | National Inpatient Sample | NR | NR | 2018 | 100 | Model 1: One-year cost (total) | Mean | USD (2020) | 6,710,047.74 | (NR) |
|  | Patients with AIS + dysphagia |  | NR | NR | 2018 | 100 | Model 1: One-year cost per patient | Mean | USD (2020) | 67,100.40 | (NR) |
|  | Patients with AIS without dysphagia |  | NR | NR | 2018 | 100 | Model 1: One-year cost (total) | Mean | USD (2020) | 5,403,130.64 | (NR) |
|  | Patients with AIS without dysphagia |  | NR | NR | 2018 | 100 | Model 1: One-year cost per patient | Mean | USD (2020) | 54,031.00 | (NR) |
|  | Patients with AIS + dysphagia vs. without dysphasia |  | NR | NR | 2018 | 100 | Model 1: Difference in one-year cost (total) | Mean | USD (2020) | 1,306,917.094 | (NR) |
|  | Patients with AIS + dysphagia vs. without dysphasia |  | NR | NR | 2018 | 100 | Model 1: Difference in one-year cost per patient | Mean | USD (2020) | 13,069.17 | (NR) |
|  | Patients with AIS + dysphagia vs. without dysphasia |  | NR | NR | 2018 | 100 | Model 1: Difference in costs for initial hospitalization | Mean | USD (2020) | 1,263,372.10 | (NR) |
|  | Patients with AIS + dysphagia | Agency for Healthcare Research and Quality, Healthcare Cost and Utilization Project; Schwarz et al. and Shireman et al. | NR | NR | 2018 | 100 | Model 2: One-year cost (total) | Mean | USD (2020) | 11,240,065.04 | (NR) |
|  | Patients with AIS + dysphagia |  | NR | NR | 2018 | 100 | Model 2: One-year cost per patient | Mean | USD (2020) | 112400.06 | (NR) |
|  | Patients with AIS without dysphagia |  | NR | NR | 2018 | 100 | Model 2: One-year cost (total) | Mean | USD (2020) | 5,197,981.28 | (NR) |
|  | Patients with AIS without dysphagia |  | NR | NR | 2018 | 100 | Model 2: One-year cost per patient | Mean | USD (2020) | 51979.80 | (NR) |
|  | Patients with AIS + dysphagia vs. without dysphasia |  | NR | NR | 2018 | 100 | Model 2: Difference in one-year cost (total) | Mean | USD (2020) | 5,702,254.65 | (NR) |
|  | Patients with AIS + dysphagia vs. without dysphasia |  | NR | NR | 2018 | 100 | Model 2: Difference in one-year cost per patient | Mean | USD (2020) | 57,022.54 | (NR) |
|  | Patients with AIS + dysphagia vs. without dysphasia |  | NR | NR | 2018 | 100 | Model 2: Difference in costs for initial hospitalization | Mean | USD (2020) | 2,949,655.68 | (NR) |
|  | Patients with AIS + dysphagia |  | NR | NR | 2018 | 352,742 | Model 1: Estimated incremental costs | Mean | USD (2020) | 4,610,038,961.13 | (NR) |
|  | Patients with AIS + dysphagia |  | NR | NR | 2018 | 352,742 | Model 2: Estimated incremental costs | Mean | USD (2020) | 20,114,218,586.23 | (NR) |
| Aggarwal, 2021(3) | Patient with AIS + acute myocardial infarction | HCUP-NIS database | 73.5 | 52.4 | 2000–2017 | 183,896 | Hospitalization costs | Mean | USD (NR) | 101,000.00 | (NR) |
| Grandhi*,* 2021(4) | Patient with AIS + EVT (Full cohort) | National Inpatient Sample | 68.7 | 50.8 | 2016–2018 | 6,052 | Total healthcare costs | Mean | USD (NR) | 184,143.00 | 0.816 |
|  | Patient with AIS + EVT (Weekend admission) |  | NR | NR | 2016–2018 | 1,660 | Total healthcare costs | Mean | USD (NR) | 183,430.00 | 0.816 |
|  | Patient with AIS + EVT (Weekday admission) |  | NR | NR | 2016–2018 | 4,392 | Total healthcare costs | Mean | USD (NR) | 184,413.00 | 0.816 |
| Khan, 2021(5) | Patients with AIS | National Inpatient Sample | NR | NR | January 2002 – December 2017 | 9,009,007 | Cumulative total inflation-adjusted cost | Mean | USD (NR) | 169.6 billion | (NR) |
|  | Patients with AIS |  | NR | NR | January 2002 – December 2017 | 9,009,007 | Inflation-adjusted cost per year | Mean | USD (NR) | 10.6 billion | (NR) |
|  | Older Patients with AIS |  | NR | NR | January 2002 – December 2017 | 6,190,274 | Cumulative total inflation-adjusted cost | Mean | USD (NR) | 105.5 billion | (NR) |
|  | Midlife Patients with AIS |  | NR | NR | January 2002 – December 2017 | 2,402,565 | Cumulative total inflation-adjusted cost | Mean | USD (NR) | 51.7 billion | (NR) |
|  | Younger Patients with AIS |  | NR | NR | January 2002 – December 2017 | 416,168 | Cumulative total inflation-adjusted cost | Mean | USD (NR) | 12.3 billion | (NR) |
| Alqahtani, 2020(6) | Patients with AIS + EVT | Vizient Clinical Database | 68.2 (15.5) | 50.3 | October 2015 – September 2019 | 22,193 | Total healthcare costs | Median | USD (2020) | 36,609.00 | (NR) |
| Wu, 2020(7) | Non-Aphasia patients with AIS | National Inpatient Sample | NR | NR | 90 Days Post-stroke | 3,604,698 | Inpatient costs | Mean | USD (NR) | 12,637.99 | <0.0001 |
|  | Aphasia patients with AIS |  | NR | NR | 90 Days Post-stroke | 734,458 | Inpatient costs | Mean | USD (NR) | 15,157.52 | <0.0001 |
| Yousufuddin, 2020(8) | Patients with AIS | Mayo  Clinic, Rochester, Minnesota | 74.9 (14.7) | 54.7 | 3 Years Post-stroke | 811 | Inpatient costs | Mean | USD (2020) | 44,347.00 | (NR) |
|  | Patients with AIS |  | 74.9 (14.7) | 54.7 | 3 Years Post-stroke | 811 | Monthly mean aggregated costs | Mean | USD (2020) | 5,138.00 | (NR) |
|  | Age <55 Patients with AIS |  | NR | NR | 3 Years Post-stroke | 92 | Inpatient costs | Mean | USD (2020) | 22,069.00 | (NR) |
|  | Age 55–64 Patients with AIS |  | NR | NR | 3 Years Post-stroke | 91 | Inpatient costs | Mean | USD (2020) | 22,538.00 | (NR) |
|  | Age 65–74 Patients with AIS |  | NR | NR | 3 Years Post-stroke | 163 | Inpatient costs | Mean | USD (2020) | 17,238.00 | (NR) |
|  | Age 75–84 Patients with AIS |  | NR | NR | 3 Years Post-stroke | 235 | Inpatient costs | Mean | USD (2020) | 17,766.00 | (NR) |
|  | Age ≥ 85 Patients with AIS |  | NR | NR | 3 Years Post-stroke | 230 | Inpatient costs | Mean | USD (2020) | 15,405.00 | (NR) |
|  | Male Patients with AIS |  | NR | NR | 3 Years Post-stroke | 367 | Inpatient costs | Mean | USD (2020) | 18,389.00 | (NR) |
|  | Female Patients with AIS |  | NR | NR | 3 Years Post-stroke | 444 | Inpatient costs | Mean | USD (2020) | 17,704.00 | (NR) |
|  | White Patients with AIS |  | NR | NR | 3 Years Post-stroke | 746 | Inpatient costs | Mean | USD (2020) | 17,276.00 | (NR) |
|  | Non-White Patients with AIS |  | NR | NR | 3 Years Post-stroke | 65 | Inpatient costs | Mean | USD (2020) | 26,485.00 | (NR) |
|  | NIHSS 0–5 Patients with AIS |  | NR | NR | 3 Years Post-stroke | 454 | Inpatient costs | Mean | USD (2020) | 15,649.00 | (NR) |
|  | NIHSS 6–10 Patients with AIS |  | NR | NR | 3 Years Post-stroke | 214 | Inpatient costs | Mean | USD (2020) | 18,147.00 | (NR) |
|  | NIHSS 11–15 Patients with AIS |  | NR | NR | 3 Years Post-stroke | 83 | Inpatient costs | Mean | USD (2020) | 27,719.00 | (NR) |
|  | NIHSS 16–20 Patients with AIS |  | NR | NR | 3 Years Post-stroke | 37 | Inpatient costs | Mean | USD (2020) | 24,373.00 | (NR) |
|  | NIHSS ≥21 Patients with AIS |  | NR | NR | 3 Years Post-stroke | 23 | Inpatient costs | Mean | USD (2020) | 18,201.00 | (NR) |
|  | Cardio embolism Patients with AIS |  | NR | NR | 3 Years Post-stroke | 230 | Inpatient costs | Mean | USD (2020) | 19,562.00 | (NR) |
|  | Patients with AIS |  | NR | NR | 3 Years Post-stroke | 30 | Inpatient costs | Mean | USD (2020) | 26,138.00 | (NR) |
|  | Large artery atherosclerosis Patients with AIS |  | NR | NR | 3 Years Post-stroke | 89 | Inpatient costs | Mean | USD (2020) | 22,710.00 | (NR) |
|  | Small vessel occlusion Patients with AIS |  | NR | NR | 3 Years Post-stroke | 200 | Inpatient costs | Mean | USD (2020) | 13,341.00 | (NR) |
|  | Patients with AIS + Hyperlipidaemia |  | NR | NR | 3 Years Post-stroke | 478 | Inpatient costs | Mean | USD (2020) | 17,797.00 | (NR) |
|  | Patients with AIS without hyperlipidaemia |  | NR | NR | 3 Years Post-stroke | 333 | Inpatient costs | Mean | USD (2020) | 18,326.00 | (NR) |
|  | Patients with AIS + Hypertension |  | NR | NR | 3 Years Post-stroke | 655 | Inpatient costs | Mean | USD (2020) | 17,929.00 | (NR) |
|  | Patients with AIS without hypertension |  | NR | NR | 3 Years Post-stroke | 156 | Inpatient costs | Mean | USD (2020) | 18,369.00 | (NR) |
|  | Patients with AIS + T2DM |  | NR | NR | 3 Years Post-stroke | 212 | Inpatient costs | Mean | USD (2020) | 17,911.00 | (NR) |
|  | Patients with AIS + No T2DM |  | NR | NR | 3 Years Post-stroke | 599 | Inpatient costs | Mean | USD (2020) | 18,050.00 | (NR) |
|  | Patients with AIS + AF |  | NR | NR | 3 Years Post-stroke | 248 | Inpatient costs | Mean | USD (2020) | 18,597.00 | (NR) |
|  | Patients with AIS + No AF |  | NR | NR | 3 Years Post-stroke | 563 | Inpatient costs | Mean | USD (2020) | 17,757.00 | (NR) |
|  | Patients with AIS + CAD |  | NR | NR | 3 Years Post-stroke | 234 | Inpatient costs | Mean | USD (2020) | 18,291.00 | (NR) |
|  | Patients with AIS + No CAD |  | NR | N | 3 Years Post-stroke | 577 | Inpatient costs | Mean | USD (2020) | 17,902.00 | (NR) |
|  | Patients with AIS + HF |  | NR | NR | 3 Years Post-stroke | 105 | Inpatient costs | Mean | USD (2020) | 20,168.00 | (NR) |
|  | Patients with AIS + No HF |  | NR | NR | 3 Years Post-stroke | 706 | Inpatient costs | Mean | USD (2020) | 17,694.00 | (NR) |
|  | Age <55 Patients with AIS |  | NR | NR | 3 Years Post-stroke | 92 | Index + 3-year monthly cost | Mean | USD (2020) | 2,411.00 | (NR) |
|  | Age 55–64 Patients with AIS |  | NR | NR | 3 Years Post-stroke | 91 | Index + 3-year monthly cost | Mean | USD (2020) | 2,883.00 | (NR) |
|  | Age 65–74 Patients with AIS |  | NR | NR | 3 Years Post-stroke | 163 | Index + 3-year monthly cost | Mean | USD (2020) | 4,227.00 | (NR) |
|  | Age 75–84 Patients with AIS |  | NR | NR | 3 Years Post-stroke | 235 | Index + 3-year monthly cost | Mean | USD (2020) | 6,500.00 | (NR) |
|  | Age ≥ 85 Patients with AIS |  | NR | NR | 3 Years Post-stroke | 230 | Index + 3-year monthly cost | Mean | USD (2020) | 6,823.00 | (NR) |
|  | Male Patients with AIS |  | NR | NR | 3 Years Post-stroke | 367 | Index + 3-year monthly cost | Mean | USD (2020) | 4,117.00 | (NR) |
|  | Female Patients with AIS |  | NR | NR | 3 Years Post-stroke | 444 | Index + 3-year monthly cost | Mean | USD (2020) | 6,214.00 | (NR) |
|  | White Patients with AIS |  | NR | NR | 3 Years Post-stroke | 746 | Index + 3-year monthly cost | Mean | USD (2020) | 5,024.00 | (NR) |
|  | Non-White Patients with AIS |  | NR | NR | 3 Years Post-stroke | 65 | Index + 3-year monthly cost | Mean | USD (2020) | 8,036.00 | (NR) |
|  | NIHSS 0–5 Patients with AIS |  | NR | NR | 3 Years Post-stroke | 454 | Index + 3-year monthly cost | Mean | USD (2020) | 2,649.00 | (NR) |
|  | NIHSS 6–10 Patients with AIS |  | NR | NR | 3 Years Post-stroke | 214 | Index + 3-year monthly cost | Mean | USD (2020) | 4,850.00 | (NR) |
|  | NIHSS 11–15 Patients with AIS |  | NR | NR | 3 Years Post-stroke | 83 | Index + 3-year monthly cost | Mean | USD (2020) | 14,635.00 | (NR) |
|  | NIHSS 16–20 Patients with AIS |  | NR | NR | 3 Years Post-stroke | 37 | Index + 3-year monthly cost | Mean | USD (2020) | 15,816.00 | (NR) |
|  | NIHSS ≥21 Patients with AIS |  | NR | NR | 3 Years Post-stroke | 23 | Index + 3-year monthly cost | Mean | USD (2020) | 9,982.00 | (NR) |
|  | Patients with AIS |  | NR | NR | 3 Years Post-stroke | 230 | Index + 3-year monthly cost | Mean | USD (2020) | 8,313.00 | (NR) |
|  | Patients with AIS |  | NR | NR | 3 Years Post-stroke | 30 | Index + 3-year monthly cost | Mean | USD (2020) | 3,118.00 | (NR) |
|  | Patients with AIS + LVO |  | NR | NR | 3 Years Post-stroke | 89 | Index + 3-year monthly cost | Mean | USD (2020) | 5,718.00 | (NR) |
|  | Patients with AIS + SVO |  | NR | NR | 3 Years Post-stroke | 200 | Index + 3-year monthly cost | Mean | USD (2020) | 2,196.00 | (NR) |
|  | Patients with AIS + Hyperlipidaemia |  | NR | NR | 3 Years Post-stroke | 478 | Index + 3-year monthly cost | Mean | USD (2020) | 3,690.00 | (NR) |
|  | Patients with AIS +No hyperlipidaemia |  | NR | NR | 3 Years Post-stroke | 333 | Index + 3-year monthly cost | Mean | USD (2020) | 7,525.00 | (NR) |
|  | Patients with AIS + Hypertension |  | NR | NR | 3 Years Post-stroke | 655 | Index + 3-year monthly cost | Mean | USD (2020) | 5,403.00 | (NR) |
|  | Patients with AIS + No hypertension |  | NR | NR | 3 Years Post-stroke | 156 | Index + 3-year monthly cost | Mean | USD (2020) | 4,684.00 | (NR) |
|  | Patients with AIS + T2DM |  | NR | NR | 3 Years Post-stroke | 212 | Index + 3-year monthly cost | Mean | USD (2020) | 5,715.00 | (NR) |
|  | Patients with AIS + No T2DM |  | NR | NR | 3 Years Post-stroke | 599 | Index + 3-year monthly cost | Mean | USD (2020) | 5,106.00 | (NR) |
|  | Patients with AIS + AF |  | NR | NR | 3 Years Post-stroke | 248 | Index + 3-year monthly cost | Mean | USD (2020) | 8,188.00 | (NR) |
|  | Patients with AIS + No AF |  | NR | NR | 3 Years Post-stroke | 563 | Index + 3-year monthly cost | Mean | USD (2020) | 3,978.00 | (NR) |
|  | Patients with AIS + CAD |  | NR | NR | 3 Years Post-stroke | 234 | Index + 3-year monthly cost | Mean | USD (2020) | 7,070.00 | (NR) |
|  | Patients with AIS + No CAD |  | NR | NR | 3 Years Post-stroke | 577 | Index + 3-year monthly cost | Mean | USD (2020) | 4,533.00 | (NR) |
|  | Patients with AIS + HF |  | NR | NR | 3 Years Post-stroke | 105 | Index + 3-year monthly cost | Mean | USD (2020) | 11,015.00 | (NR) |
|  | Patients with AIS + No HF |  | NR | NR | 3 Years Post-stroke | 706 | Index + 3-year monthly cost | Mean | USD (2020) | 4,410.00 | (NR) |
| Alkhouli, 2018(9) | Patients with AIS without AF (unmatched cohorts) | National Inpatient Sample | 70 (15) | 51.8 | 2003–2014 | 761,204 | Inpatient costs | Median | USD (NR) | 7,546.00 | (NR) |
|  | Patients with AIS + AF (unmatched cohorts) |  | 82 (10) | 59.3 | 2003–2014 | 168,806 | Inpatient costs | Median | USD (NR) | 8,683.00 | <0.001 |
|  | Patients with AIS without AF (matched cohorts) |  | 79 (11) | 58.7 | 2003–2014 | 125,203 | Inpatient costs | Median | USD (NR) | 8,127.00 | (NR) |
|  | Patients with AIS + AF (matched cohorts) |  | 79 (10) | 58.7 | 2003–2014 | 125,203 | Inpatient costs | Median | USD (NR) | 9,475.00 | <0.001 |
| Dave, 2018(10) | Patients admitted to Neurology services for AIS | Military Health System, Military Mart (M2) administrative database | 69.4 | 40.3 | 2010–2015 | 462 | Total healthcare costs | Mean | USD (NR) | 13,622.0.00 | <0.005 |
|  | Patients admitted to Primary care services for AIS |  | 67.1 | 38.7 | 2010–2015 | 2,324 | Total healthcare costs | Mean | USD (NR) | 12,549.00 | <0.005 |
|  | Patients admitted to ICUs for AIS |  | 62.1 | 40 | 2010–2015 | 677 | Total healthcare costs | Mean | USD (NR) | 30,622.00 | (NR) |
| Mu, 2017(11) | Patients with AIS | IMS LifeLink PharMetrics Plus administrative claims database | 59.7 | 47.7 | 360 Days Post-stroke | 207,392 | Third-party costs during index stroke hospitalization | Mean | USD (2015) | 31,667.00 | (NR) |
|  | Dead at discharge patients |  | NR | NR | 360 Days Post-stroke | 7,919 | Third-party costs during index stroke hospitalization | Mean | USD (2015) | 63,605.00 | <0.001 |
|  | Discharged with disability patients |  | NR | NR | 360 Days Post-stroke | 45,695 | Third-party costs during index stroke hospitalization | Mean | USD (2015) | 67,861.00 | <0.001 |
|  | Discharged without disability patients |  | NR | NR | 360 Days Post-stroke | 153,778 | Third-party costs during index stroke hospitalization | Mean | USD (2015) | 19,267.00 | <0.001 |
|  | Patients with AIS |  | NR | NR | 360 Days Post-stroke | 207,392 | Out-of-pocket costs during index stroke hospitalization | Mean | USD (2015) | 5,355.00 | (NR) |
|  | Dead at discharge patients |  | NR | NR | 360 Days Post-stroke | 7,919 | Out-of-pocket costs during index stroke hospitalization | Mean | USD (2015) | 4,766.00 | <0.001 |
|  | Discharged with disability patients |  | NR | NR | 360 Days Post-stroke | 45,695 | Out-of-pocket costs during index stroke hospitalization | Mean | USD (2015) | 6,041.00 | <0.001 |
|  | Discharged without disability patients |  | NR | NR | 360 Days Post-stroke | 153,778 | Out-of-pocket costs during index stroke hospitalization | Mean | USD (2015) | 5,181.00 | <0.001 |
|  | Patients with AIS |  | NR | NR | 360 Days Post-stroke | 113,474 | Third-party costs during index stroke hospitalization | Mean | USD (2015) | 29,364.00 | (NR) |
|  | Discharged with disability patients |  | NR | NR | 360 Days Post-stroke | 20,417 | Third-party costs during index stroke hospitalization | Mean | USD (2015) | 41,565.00 | <0.001 |
|  | Discharged without disability patients |  | NR | NR | 360 Days Post-stroke | 93,057 | Third-party costs during index stroke hospitalization | Mean | USD (2015) | 26,687.00 | <0.001 |
|  | Patients with AIS |  | NR | NR | 360 Days Post-stroke | 113,474 | Out-of-pocket costs during index stroke hospitalization | Mean | USD (2015) | 3,776.00 | (NR) |
|  | Discharged with disability patients |  | NR | NR | 360 Days Post-stroke | 20,417 | Out-of-pocket costs during index stroke hospitalization | Mean | USD (2015) | 5,285.00 | <0.001 |
|  | Discharged without disability patients |  | NR | NR | 360 Days Post-stroke | 93,057 | Out-of-pocket costs during index stroke hospitalization | Mean | USD (2015) | 3,445.00 | <0.001 |
|  | Patients with AIS |  | NR | NR | 360 Days Post-stroke | 113,474 | Inpatient costs | Mean | USD (2015) | 12,778.00 | <0.05 |
|  | Patients with AIS |  | NR | NR | 360 Days Post-stroke | 113,474 | Outpatient costs | Mean | USD (2015) | 10,108.00 | <0.05 |
|  | Patients with AIS |  | NR | NR | 360 Days Post-stroke | 113,474 | Emergency room | Mean | USD (2015) | 838.00 | <0.05 |
|  | Patients with AIS |  | NR | NR | 360 Days Post-stroke | 113,474 | Other medical | Mean | USD (2015) | 2,048.00 | <0.05 |
|  | Patients with AIS |  | NR | NR | 360 Days Post-stroke | 113,474 | Pharmacy | Mean | USD (2015) | 3,592.00 | <0.05 |
|  | Discharged with disability patients |  | NR | NR | 360 Days Post-stroke | 20,417 | Inpatient costs | Mean | USD (2015) | 20,140.00 | <0.05 |
|  | Discharged with disability patients |  | NR | NR | 360 Days Post-stroke | 20,417 | Outpatient costs | Mean | USD (2015) | 12,264.00 | <0.05 |
|  | Discharged with disability patients |  | NR | NR | 360 Days Post-stroke | 20,417 | Emergency room | Mean | USD (2015) | 953.00 | <0.05 |
|  | Discharged with disability patients |  | NR | NR | 360 Days Post-stroke | 20,417 | Other medical | Mean | USD (2015) | 4,513.00 | <0.05 |
|  | Discharged with disability patients |  | NR | NR | 360 Days Post-stroke | 20,417 | Pharmacy | Mean | USD (2015) | 3,696.00 | <0.05 |
|  | Discharged without disability patients |  | NR | NR | 360 Days Post-stroke | 93,057 | Inpatient costs | Mean | USD (2015) | 11,162.00 | <0.05 |
|  | Discharged without disability patients |  | NR | NR | 360 Days Post-stroke | 93,057 | Outpatient costs | Mean | USD (2015) | 9,635.00 | <0.05 |
|  | Discharged without disability patients |  | NR | NR | 360 Days Post-stroke | 93,057 | Emergency room | Mean | USD (2015) | 812.00 | <0.05 |
|  | Discharged without disability patients |  | NR | NR | 360 Days Post-stroke | 93,057 | Other medical | Mean | USD (2015) | 1,508.00 | <0.05 |
|  | Discharged without disability patients |  | NR | NR | 360 Days Post-stroke | 93,057 | Pharmacy | Mean | USD (2015) | 3,571.00 | <0.05 |
| Mukherjee*,* 2019(12) | Patients with AIS | National Inpatient Sample | NR | 50.35 | 2010–2013 | 434,544 | Inpatient costs | Mean | USD (NR) | 13,072.00 | (NR) |
|  | Patients with AIS |  |  |  | 2010–2013 | 434,544 | Inpatient costs | Median | USD (NR) | 9,270.87 | (NR) |
|  | Patients with AIS + AF |  | 78.9 | 56.38 | 2010–2013 | 90,190 | Inpatient costs | Mean | USD (NR) | 15,112.00 | (NR) |
|  | Patients with AIS + AF |  |  |  | 2010–2013 | 90,190 | Inpatient costs | Median | USD (NR) | 10,420.00 | (NR) |
|  | Patients with AIS without AF |  | 68.81 | 48.77 | 2010–2013 | 344,354 | Inpatient costs | Mean | USD (NR) | 12,538.00 | (NR) |
|  | Patients with AIS without AF |  |  |  | 2010–2013 | 344,354 | Inpatient costs | Median | USD (NR) | 9,021.00 | (NR) |
| Chaudhry*,* 2016(13) | Patients admitted to primary stroke centre | National Inpatient Sample | NR | NR | 2010–2011 | 72,982 | Inpatient costs | Mean | USD (NR) | 47,621.00 | <.0001 |
|  | Patients admitted to non-primary centre |  | NR | NR | 2010–2011 | 50,149 | Inpatient costs | Mean | USD (NR) | 35,229.00 | <.0001 |
| Johnson, 2016(14) | Commercial patients with AIS | MarketScan® Commercial Claims and Encounters Database and MarketScan® Medicare Supplemental and Coordination of Benefits | 54.1 (NR) | 42.1 | 1-Year post-stroke | 20,314 | Total costs | Mean | USD (2013) | 61,354.00 | (NR) |
|  | Medicare patients with AIS |  | 78.6 (NR) | 53.1 | 1-Year post-stroke | 31,037 | Total costs | Mean | USD (2013) | 44,929.00 | (NR) |
|  | Commercial patients with AIS |  | NR | NR | 0–30 Days Post-stroke | 20,314 | Total costs | Mean | USD (2013) | 19,682.00 | (NR) |
|  | Medicare patients with AIS |  | NR | NR | 0–30 Days Post-stroke | 31,037 | Total costs | Mean | USD (2013) | 14,981.00 | (NR) |
|  | Commercial patients with AIS |  | NR | NR | 31–365 Days Post-stroke | 20,314 | Total costs | Mean | USD (2013) | 5,055.00 | (NR) |
|  | Medicare patients with AIS |  | NR | NR | 31–365 Days Post-stroke | 31,037 | Total costs | Mean | USD (2013) | 5,060.00 | (NR) |
|  | Commercial patients with AIS |  | NR | NR | 1-Year post-stroke | 20,314 | Total costs | Mean | USD (2013) | 24,737.00 | (NR) |
|  | Medicare patients with AIS |  | NR | NR | 1-Year post-stroke | 31,037 | Total costs | Mean | USD (2013) | 20,041.00 | (NR) |
|  | Commercial patients with major complications/comorbidities |  | NR | NR | 1-Year post-stroke | 6,396 | Total costs | Mean | USD (2013) | 29,933.00 | (NR) |
|  | Commercial patients with complications/comorbidities |  | NR | NR | 1-Year post-stroke | 9,784 | Total costs | Mean | USD (2013) | 23,933.00 | (NR) |
|  | Commercial patients without complications/comorbidities |  | NR | NR | 1-Year post-stroke | 4,134 | Total costs | Mean | USD (2013) | 18,601.00 | (NR) |
|  | Medicare patients with major complications/comorbidities |  | NR | NR | 1-Year post-stroke | 9,939 | Total costs | Mean | USD (2013) | 23,260.00 | (NR) |
|  | Medicare patients with complications/comorbidities |  | NR | NR | 1-Year post-stroke | 14,831 | Total costs | Mean | USD (2013) | 20,261.00 | (NR) |
|  | Medicare patients without complications/comorbidities |  | NR | NR | 1-Year post-stroke | 6,567 | Total costs | Mean | USD (2013) | 14,818.00 | (NR) |
| Kumar, 2016(15) | Patients with AIS | National Inpatient Sample | 73 | 52.7 | 2011–2012 | 835,811 | Inpatient hospitalization costs | Mean | USD (2012) | 43,362.00 | (NR) |
| Sonig*,* 2016(16) | Directly admitted patients with AIS | National Inpatient Sample | 71.11 | 50.7 | 2008–2010 | 55,913 | Inpatient hospitalization costs | Mean | USD (NR) | 70,325.11 | (NR) |
|  | Patients transferred from another facility |  |  |  | 2008–2010 | 55,913 | Inpatient hospitalization costs | Mean | USD (NR) | 97,546.92 | (NR) |
| Tong, 2016(17) | Age 85+ Patients with AIS | National Inpatient Sample | NR | NR | 2-Year (2003–2004) | 172,992 | Total costs | Mean | USD (2012) | 9,373.00 | (NR) |
|  | Age 18–34 Patients with AIS |  | NR | NR | 2-Year (2011–2012) | 10,178 | Total costs | Mean | USD (2012) | 15,967.00 | (NR) |
|  | Age 35–44 Patients with AIS |  | NR | NR | 2-Year (2011–2012) | 28,287 | Total costs | Mean | USD (2012) | 14,379.00 | (NR) |
|  | Age 45–54 Patients with AIS |  | NR | NR | 2-Year (2011–2012) | 102,083 | Total costs | Mean | USD (2012) | 12,976.00 | (NR) |
|  | Age 55–64 Patients with AIS |  | NR | NR | 2-Year (2011–2012) | 202,227 | Total costs | Mean | USD (2012) | 12,374.00 | (NR) |
|  | Age 65–74 Patients with AIS |  | NR | NR | 2-Year (2011–2012) | 249,585 | Total costs | Mean | USD (2012) | 11,337.00 | (NR) |
|  | Age 75–84 Patients with AIS |  | NR | NR | 2-Year (2011–2012) | 298,210 | Total costs | Mean | USD (2012) | 10,942.00 | (NR) |
|  | Age 85+ Patients with AIS |  | NR | NR | 2-Year (2011–2012) | 198,904 | Total costs | Mean | USD (2012) | 10,368.00 | (NR) |
|  | Patients with AIS |  | NR | NR | 2-Year (2003–2004) | (NR) | Total costs in billions | Mean | USD (2012) | 10.67 | (NR) |
|  | Patients with AIS |  | NR | NR | 2-Year (2011–2012) | (NR) | Total costs in billions | Mean | USD (2012) | 12.55 | (NR) |
| Agarwal, 2015(18) | SES Quartile 1 (Median household income $1 to 38,999) | National Inpatient Sample | NR | NR | 9-Year (2003–2011) | 230,028 | Inpatient hospitalization costs | Mean | USD (2011) | 11,672.00 | (NR) |
|  | SES Quartile 2 (Median household income $39,000 to 47,999) |  | NR | NR | 9-Year (2003–2011) | 206,523 | Inpatient hospitalization costs | Mean | USD (2011) | 11,666.00 | (NR) |
|  | SES Quartile 3 (Median household income $48,000 to 62,999) |  | NR | NR | 9-Year (2003–2011) | 182,991 | Inpatient hospitalization costs | Mean | USD (2011) | 12,579.00 | (NR) |
|  | SES Quartile 4 (Median household income $≥63 000) |  | NR | NR | 9-Year (2003–2011) | 156,363 | Inpatient hospitalization costs | Mean | USD (2011) | 14,195.00 | (NR) |
| Pan, 2015(19) | Patients with AIS + AF vs. non-AF | Premier Alliance Database | 70.29 | NR | January 2006 – June 2011 | 351,601 | Modelled inpatient costs | Cost difference | USD (NR) | 2,997.18 | <0.0001 |
|  | Patients with AIS Age: 18-39 vs. 80+ |  | 70.29 | NR | January 2006 – June 2011 | 351,601 | Modelled inpatient costs | Cost difference | USD (NR) | 4,542.61 | <0.0001 |
|  | Patients with AIS Age: 40-49 vs. 80+ |  | 70.29 | NR | January 2006 – June 2011 | 351,601 | Modelled inpatient costs | Cost difference | USD (NR) | 2,651.13 | <0.0001 |
|  | Patients with AIS Age: 50-59 vs. 80+ |  | 70.29 | NR | January 2006 – June 2011 | 351,601 | Modelled inpatient costs | Cost difference | USD (NR) | 2,009.55 | <0.0001 |
|  | Patients with AIS Age: 60-69 vs. 80+ |  | 70.29 | NR | January 2006 – June 2011 | 351,601 | Modelled inpatient costs | Cost difference | USD (NR) | 1,235.46 | <0.0001 |
|  | Patients with AIS Age: 70-79 vs. 80+ |  | 70.29 | NR | January 2006 – June 2011 | 351,601 | Modelled inpatient costs | Cost difference | USD (NR) | 745.56 | <0.0001 |
|  | Patients with AIS Sex: Male vs. female |  | 70.29 | N | January 2006 – June 2011 | 351,601 | Modelled inpatient costs | Cost difference | USD (NR) | 308.80 | <0.0001 |
|  | Patients with AIS + M vs. no MI |  | 70.29 | NR | January 2006 – June 2011 | 351,601 | Modelled inpatient costs | Cost difference | USD (NR) | 1,228.62 | <0.0001 |
|  | Patients with AIS + Congestive HF vs. no HF |  | 70.29 | NR | January 2006 – June 2011 | 351,601 | Modelled inpatient costs | Cost difference | USD (NR) | 2,338.77 | <0.0001 |
|  | Patients with AIS + T2DM vs. no T2DM |  | 70.29 | NR | January 2006 – June 2011 | 351,601 | Modelled inpatient costs | Cost difference | USD (NR) | 473.68 | <0.0001 |
|  | Patients with AIS + Hypertension vs. no hypertension |  | 70.29 | NR | January 2006 – June 2011 | 351,601 | Modelled inpatient costs | Cost difference | USD (NR) | -536.92 | <0.0001 |
|  | Patients with AIS Race: Hispanic vs. Black |  | 70.29 | NR | January 2006 – June 2011 | 351,601 | Modelled inpatient costs | Cost difference | USD (NR) | 1,188.85 | <0.0001 |
|  | Patients with AIS Race: Other vs. Black |  | 70.29 | NR | January 2006 – June 2011 | 351,601 | Modelled inpatient costs | Cost difference | USD (NR) | -591.89 | <0.0001 |
|  | Patients with AIS Race: White vs. Black |  | 70.29 | NR | January 2006 – June 2011 | 351,601 | Modelled inpatient costs | Cost difference | USD (NR) | -1,087.18 | <0.0001 |
| **China** | | | | | | | | | | | |
| Su*,* 2022(20) | Patient with AIS | Nationwide Inpatient Sample | NR | NR | 2015–2020 | 129,444 | Hospitalization costs discounted by the CPI, taking 2020 as the benchmark year. | Median | USD (2020) | 1,020.00 | (NR) |
|  | Patient with AIS |  | NR | NR | 2015–2020 | 129,444 | Hospitalization costs discounted by the CPI, taking 2020 as the benchmark year. | Median | USD (2020) | 936.00 | (NR) |
|  | Patient with AIS |  | NR | NR | 2015–2020 | 129,444 | Hospitalization costs discounted by the CPI, taking 2020 as the benchmark year. | Median | USD (2015) | 1,181.00 | (NR) |
|  | Patient with AIS |  | NR | NR | 2015–2020 | 129,444 | Hospitalization costs discounted by the CPI, taking 2020 as the benchmark year. | Median | USD (2016) | 1,294.00 | (NR) |
|  | Patient with AIS |  | NR | NR | 2015–2020 | 129,444 | Hospitalization costs discounted by the CPI, taking 2020 as the benchmark year. | Median | USD (2017) | 1,076.00 | (NR) |
|  | Patient with AIS |  | NR | NR | 2015–2020 | 129,444 | Hospitalization costs discounted by the CPI, taking 2020 as the benchmark year. | Median | USD (2018) | 940.00 | (NR) |
|  | Patient with AIS |  | NR | NR | 2015–2020 | 129,444 | Hospitalization costs discounted by the CPI, taking 2020 as the benchmark year. | Median | USD (2019) | 967.00 | (NR) |
|  | Patient with AIS |  | NR | NR | 2015–2020 | 129,444 | Hospitalization costs discounted by the CPI, taking 2020 as the benchmark year. | Median | USD (2020) | 936.0 | (NR) |
| Duan *et al*. 2021(21) | Patient with AIS | Registry data from the China Stroke Center Alliance (CSCA) | 66 | 37.8 | August 2015 –July 2019 | 893,429 | Hospitalization costs | Mean | RMB (NR) | 12,372.40 | (NR) |
|  | Patient with AIS |  | 66 | 37.8 | August 2015 –July 2019 | 893,429 | Medicine expenditure | Mean | RMB (NR) | 5,467.80 | (NR) |
|  | Patient with AIS + acute MI |  | 70 | 43.9 | August 2015 –July 2019 | 81,646 | Hospitalization costs | Mean | RMB (NR) | 13,342.90 | (NR) |
|  | Patient with AIS + acute MI |  | 70 | 43.9 | August 2015 –July 2019 | 81,646 | Medicine expenditure | Mean | RMB (NR) | 6,045.10 | (NR) |
|  | Patient with AIS + acute MI |  | 65.6 | 37.2 | August 2015 –July 2019 | 811,783 | Hospitalization costs | Mean | RMB (NR) | 12,275.10 | (NR) |
|  | Patient with AIS + acute MI |  | 65.6 | 37.2 | August 2015 –July 2019 | 811,783 | Medicine expenditure | Mean | RMB (NR) | 5,409.70 | (NR) |
| Lu, 2021(22) | Patients with AIS: Eastern area | Chinese Acute Ischemic Stroke Treatment Outcome Registry (CASTOR), data from 80 hospitals in 44 cities across mainland China. | 64 | 34.4 | 2015–2017 | 8,547 | Total healthcare costs | Median | USD (2017) | 2,175.90 | <0.001 |
|  | Patients with AIS: Northeastern area |  | 64 | 34.4 | 2015–2017 | 8,547 | Total healthcare costs | Median | USD (2017) | 2,175.10 | <0.001 |
|  | Patients with AIS: Central area |  | 64 | 34.4 | 2015–2017 | 8,547 | Total healthcare costs | Median | USD (2017) | 2,477.70 | <0.001 |
|  | Patients with AIS: Western area |  | 64 | 34.4 | 2015–2017 | 8,547 | Total healthcare costs | Median | USD (2017) | 2,282.40 | <0.001 |
| Xin, 2021(23) | Patients with AIS admitted in 2019 | Fujian Medical University Union Hospital database | 69 | 47 | January –February 2020 | 38 | Total healthcare costs | Median | CNY (NR) | 12,084.00 | 0.231 |
|  | Patients with AIS admitted in 2020 |  | 67 | 22 | January –February 2020 | 37 | Total healthcare costs | Median | CNY (NR) | 14,423.00 | 0.231 |
| Liu, 2020(24) | Immobile patients with AIS | Standardized Nursing Intervention Model study (SNIM) | NR | NR | November 2015 – July 2016 | 3,143 | Inpatient costs (Materials fee) | Mean | RMB (2016) | 6,836.92 | (NR) |
|  | Immobile patients with AIS |  | NR | NR | November 2015 – July 2016 | 3,143 | Inpatient costs (Medical service fee) | Mean | RMB (2016) | 5,667.42 | (NR) |
|  | Immobile patients with AIS |  | NR | NR | November 2015 – July 2016 | 3,143 | Inpatient costs (Lab/ examination fee) | Mean | RMB (2016) | 5,170.69 | (NR) |
|  | Immobile patients with AIS |  | NR | NR | November 2015 – July 2016 | 3,143 | Inpatient costs (Nursing fee) | Mean | RMB (2016) | 537.88 | (NR) |
|  | Immobile patients with AIS |  | NR | NR | November 2015 – July 2016 | 3,143 | Inpatient costs (Others) | Mean | RMB (2016) | 177.91 | (NR) |
|  | Immobile patients with AIS |  | NR | NR | November 2015 – July 2016 | 3,143 | Inpatient costs (Blood transfusion fee) | Mean | RMB (2016) | 122.24 | (NR) |
|  | Immobile patients with AIS |  | NR | NR | November 2015 – July 2016 | 3,143 | Inpatient costs (Materials fee) | Median | RMB (2016) | 238.05 | (NR) |
|  | Immobile patients with AIS |  | NR | NR | November 2015 – July 2016 | 3,143 | Inpatient costs (Medical service fee) | Median | RMB (2016) | 2,922.60 | (NR) |
|  | Immobile patients with AIS |  | NR | NR | November 2015 – July 2016 | 3,143 | Inpatient costs (Lab/ examination fee) | Median | RMB (2016) | 3,468.00 | (NR) |
|  | Immobile patients with AIS |  | NR | NR | November 2015 – July 2016 | 3,143 | Inpatient costs (Nursing fee) | Median | RMB (2016) | 212.00 | (NR) |
|  | Immobile patients with AIS |  | NR | NR | November 2015 – July 2016 | 3,143 | Inpatient costs (others) | Median | RMB (2016) | 0.00 | (NR) |
|  | Immobile patients with AIS |  | NR | NR | November 2015 – July 2016 | 3,143 | Inpatient costs (Blood transfusion fee) | Median | RMB (2016) | 0.00 | (NR) |
| Yang, 2020(25) | Male patients with AIS | China Urban Employees’ Basic Medical Insurance (UEBMI) and Urban Residents’ Basic Medical Insurance (URBMI) | NR | NR | 2015 | 32,658 | Hospitalization costs | Mean | RMB (2015) | 10 044.32 | <0.001 |
|  | Female patients with AIS |  | NR | NR | 2015 | 23,827 | Hospitalization costs | Mean | RMB (2015) | 8,738.35 | <0.001 |
|  | Age 0–44 patients with AIS |  | NR | NR | 2015 | 1,222 | Hospitalization costs | Mean | RMB (2015) | 10 957.67 | <0.001 |
|  | Age 44–59 patients with AIS |  | NR | NR | 2015 | 10,825 | Hospitalization costs | Mean | RMB (2015) | 8,926.78 | <0.001 |
|  | Age ≥60 patients with AIS |  | NR | NR | 2015 | 44,438 | Hospitalization costs | Mean | RMB (2015) | 9,591.19 | <0.001 |
|  | ICD Code I63 Patients with AIS |  | NR | NR | 2015 | 472 | Hospitalization costs | Mean | RMB (2015) | 14 618.74 | 0.869 |
|  | ICD Code I63.0 Patients with AIS |  | NR | NR | 2015 | 5 | Hospitalization costs | Mean | RMB (2015) | 6,072.54 | 0.683 |
|  | ICD Code I63.1 Patients with AIS |  | NR | NR | 2015 | 4 | Hospitalization costs | Mean | RMB (2015) | 3,190.43 | 0.965 |
|  | ICD Code I63.2 Patients with AIS |  | NR | NR | 2015 | 14 | Hospitalization costs | Mean | RMB (2015) | 22 449.77 | 0.356 |
|  | ICD Code I63.3 Patients with AIS |  | NR | NR | 2015 | 41 | Hospitalization costs | Mean | RMB (2015) | 19 720.70 | 0.180 |
|  | ICD Code I63.4 Patients with AIS |  | NR | NR | 2015 | 45 | Hospitalization costs | Mean | RMB (2015) | 7,444.69 | <0.001 |
|  | ICD Code I63.5 Patients with AIS |  | NR | NR | 2015 | 54 | Hospitalization costs | Mean | RMB (2015) | 4,224.79 | <0.001 |
|  | ICD Code I63.8 Patients with AIS |  | NR | NR | 2015 | 78 | Hospitalization costs | Mean | RMB (2015) | 15 161.85 | 0.302 |
|  | ICD Code I63.9 Patients with AIS |  | NR | NR | 2015 | 55,772 | Hospitalization costs | Mean | RMB (2015) | 9,438.86 | <0.001 |
|  | Primary Hospital Patients with AIS |  | NR | NR | 2015 | 11,110 | Hospitalization costs | Mean | RMB (2015) | 4,297.96 | <0.001 |
|  | Secondary Hospital Patients with AIS |  | NR | NR | 2015 | 26,932 | Hospitalization costs | Mean | RMB (2015) | 7,776.65 | <0.001 |
|  | Tertiary Hospital Patients with AIS |  | NR | NR | 2015 | 18,443 | Hospitalization costs | Mean | RMB (2015) | 15 130.12 | <0.001 |
|  | Patients with AIS: East |  | NR | NR | 2015 | 20,850 | Hospitalization costs | Mean | RMB (2015) | 11 881.32 | <0.001 |
|  | Patients with AIS: Central |  | NR | NR | 2015 | 26,184 | Hospitalization costs | Mean | RMB (2015) | 7,836.13 | <0.001 |
|  | Patients with AIS: West |  | NR | NR | 2015 | 9,451 | Hospitalization costs | Mean | RMB (2015) | 8,816.99 | <0.001 |
| Kong*,* 2018(26) | Patients with AIS | Beijing Public Health Information Center | 67.7 | NR | March 2012 –February 2015 | 121,373 | Total healthcare costs | Mean | USD (NR) | 2,812.00 | <0.001 |
|  | Patients with AIS |  | 67.7 | NR | March 2012 –February 2015 | 121,373 | Total healthcare costs | Median | USD (NR) | 2,109.00 | <0.001 |
|  | Patients with AIS |  | 67.7 | NR | March 2012 –February 2015 | 121,373 | Therapy overall fee | Median | USD (NR) | 135.00 | <0.001 |
|  | Patients with AIS |  | 67.7 | NR | March 2012 –February 2015 | 121,373 | Rehabilitation therapy cost | Median | USD (NR) | 0.00 | 0.002 |
|  | Patients with AIS |  | 67.7 | NR | March 2012 –February 2015 | 121,373 | Oxygen cost | Median | USD (NR) | 11.00 | <0.001 |
|  | Patients with AIS |  | 67.7 | NR | March 2012 –February 2015 | 121,373 | Monitoring and assisted respiration cost | Median | USD (NR) | 0.00 | <0.001 |
|  | Patients with AIS |  | 67.7 | NR | March 2012 –February 2015 | 121,373 | Nursing treatment cost | Median | USD (NR) | 24.00 | <0.001 |
|  | Patients with AIS |  | 67.7 | NR | March 2012 –February 2015 | 121,373 | Lab and examination overall fee | Median | USD (NR) | 542.00 | <0.001 |
|  | Patients with AIS |  | 67.7 | NR | March 2012 –February 2015 | 121,373 | Ultrasound cost | Median | USD (NR) | 86.00 | <0.001 |
|  | Patients with AIS |  | 67.7 | NR | March 2012 –February 2015 | 121,373 | Imaging cost | Median | USD (NR) | 140.00 | <0.001 |
|  | Patients with AIS |  | 67.7 | NR | March 2012 –February 2015 | 121,373 | Laboratory cost | Median | USD (NR) | 258.00 | <0.001 |
|  | Patients with AIS |  | 67.7 | NR | March 2012 –February 2015 | 121,373 | Medical disposable material fee | Median | USD (NR) | 116.0 | <0.001 |
|  | Patients with AIS |  | 67.7 | NR | March 2012 –February 2015 | 121,373 | Overall medicine fee | Median | USD (NR) | 1,026.00 | <0.001 |
|  | Patients with AIS |  | 67.7 | NR | March 2012 –February 2015 | 121,373 | Total costs (Western medicine fee) | Median | USD (NR) | 893.00 | <0.001 |
|  | Patients with AIS |  | 67.7 | NR | March 2012 –February 2015 | 121,373 | Antibiotic medicine costs | Median | USD (NR) | 0.00 | <0.001 |
|  | Patients with AIS |  | 67.7 | NR | March 2012 –February 2015 | 121,374 | Hospitalization costs | Median | USD (NR) | 51.00 | <0.001 |
| Wen, 2017(27) | Patients with AIS + AF | Patient insurance claims from the Beijing Urban Medical Insurance database | 73.82 (11.72) | 41 | January –December 2012 | 992 | Total costs | Mean | RMB (NR) | 163550.40 | (NR) |
|  | Patients with AIS without AF |  | 66.71 (14.18) | 35 | January –December 2012 | 3,069 | Total costs | Mean | RMB (NR) | 64735.41 | (NR) |
|  | Patients with AIS + AF |  | 73.82 (11.72) | 41 | January –December 2012 | 992 | Inpatient cost per patient in the observational period | Mean | RMB (NR) | 141875.90 | (NR) |
|  | Patients with AIS without AF |  | 66.71 (14.18) | 35 | January –December 2012 | 3,069 | Inpatient cost per patient in the observational period | Mean | RMB (NR) | 53834.03 | (NR) |
|  | Patients with AIS + AF |  | 73.82 (11.72) | 41 | January –December 2012 | 992 | Outpatient cost per patient in the observational period | Mean | RMB (NR) | 21674.46 | (NR) |
|  | Patients with AIS without AF |  | 66.71 (14.18) | 35 | January –December 2012 | 3,069 | Outpatient cost per patient in the observational period | Mean | RMB (NR) | 10901.38 | (NR) |
|  | Patients with AIS + AF |  | 73.82 (11.72) | 41 | January –December 2012 | 992 | Total healthcare cost covered by medical insurance | Mean | RMB (NR) | 132,036.70 | (NR) |
|  | Patients with AIS without AF |  | 66.71 (14.18) | 35 | January –December 2012 | 3,069 | Total healthcare cost covered by medical insurance | Mean | RMB (NR) | 50,379.90 | (NR) |
|  | Patients with AIS + AF |  | 73.82 (11.72) | 41 | January –December 2012 | 992 | Inpatient costs | Mean | RMB (NR) | 167,644.20 | (NR) |
|  | Patients with AIS without AF |  | 66.71 (14.18) | 35 | January –December 2012 | 3,069 | Inpatient costs | Mean | RMB (NR) | 72,558.26 | (NR) |
| Li, 2017(28) | Patients with AIS + normal sinus rhythm / AF | Dept of Neurology, Huizhou Municipal Central Hospital, Huizhou Hospital Affiliated to Guangdong Medical College | 64.7 | 36.2 | 2014 | 824 | Total costs | Mean | CNY→USD (2014) | 1,247.60 | 0.001 |
|  | Patients with AIS + normal sinus rhythm / AF |  | 64.7 | 36.2 | 2014 | 824 | Total costs | Mean | CNY→USD (2014) | 71.46 | 0.001 |
|  | Patients with AIS + normal sinus rhythm / AF |  | 64.7 | 36.2 | 2014 | 824 | Total costs | Mean | CNY→USD (2014) | 6.63 | <0.001 |
|  | Patients with AIS + normal sinus rhythm / AF |  | 64.7 | 36.2 | 2014 | 824 | Total costs | Mean | CNY→USD (2014) | 42.94 | <0.001 |
|  | Patients with AIS + normal sinus rhythm / AF |  | 64.7 | 36.2 | 2014 | 824 | Total costs | Mean | CNY→USD (2014) | 15.08 | 0.792 |
|  | Patients with AIS + normal sinus rhythm / AF |  | 64.7 | 36.2 | 2014 | 824 | Total costs | Mean | CNY→USD (2014) | 75.16 | 0.241 |
|  | Patients with AIS + normal sinus rhythm / AF |  | 64.7 | 36.2 | 2014 | 824 | Total costs | Mean | CNY→USD (2014) | 7.35 | <0.001 |
|  | Patients with AIS + normal sinus rhythm / AF |  | 64.7 | 36.2 | 2014 | 824 | Total costs | Mean | CNY→USD (2014) | 20.41 | <0.001 |
|  | Patients with AIS + normal sinus rhythm / AF |  | 64.7 | 36.2 | 2014 | 824 | Total costs | Mean | CNY→USD (2014) | 208.84 | <.001 |
|  | Patients with AIS + normal sinus rhythm / AF |  | 64.7 | 36.2 | 2014 | 824 | Total costs | Mean | CNY→USD (2014) | 280.88 | <0.001 |
|  | Patients with AIS + normal sinus rhythm / AF |  | 64.7 | 36.2 | 2014 | 824 | Total costs | Mean | CNY→USD (2014) | 2.16 | 0.035 |
|  | Patients with AIS + normal sinus rhythm / AF |  | 64.7 | 36.2 | 2014 | 824 | Total costs | Mean | CNY→USD (2014) | 216.68 | 0.631 |
|  | Patients with AIS + normal sinus rhythm / AF |  | 64.7 | 36.2 | 2014 | 824 | Total costs | Mean | CNY→USD (2014) | 140.54 | 0.205 |
|  | Patients with AIS + normal sinus rhythm / AF |  | 64.7 | 36.2 | 2014 | 824 | Total costs | Mean | CNY→USD (2014) | 6.24 | 0.322 |
|  | Patients with AIS + normal sinus rhythm / AF |  | 64.7 | 36.2 | 2014 | 824 | Total costs | Mean | CNY→USD (2014) | 2,341.98 | <0.001 |
| **South Korea** | | | | | | | | | | | |
| Kim, 2020(29) | Patients with AIS: Previous year | Clinical Research Collaboration for Stroke in Korea | >65 years | 42 | 5-Years post-stroke | 11,136 | Annual and 5-year cumulative costs according to 3-month mRS score | Mean | USD (2016) | 8,718.00 | (NR) |
|  | Patients with AIS: First year |  | >65 years | 42 | 5-Years post-stroke | 11,136 | Annual and 5-year cumulative costs according to 3-month mRS score | Mean | USD (2016) | 38,152.00 | (NR) |
|  | Patients with AIS: Second year |  | >65 years | 42 | 5-Years post-stroke | 11,136 | Annual and 5-year cumulative costs according to 3-month mRS score | Mean | USD (2016) | 22,094.00 | (NR) |
|  | Patients with AIS: Third year |  | >65 years | 42 | 5-Years post-stroke | 11,136 | Annual and 5-year cumulative costs according to 3-month mRS score | Mean | USD (2016) | 20,634.00 | (NR) |
|  | Patients with AIS: Fourth year |  | >65 years | 42 | 5-Years post-stroke | 7,361 | Annual and 5-year cumulative costs according to 3-month mRS score | Mean | USD (2016) | 18,757.00 | (NR) |
|  | Patients with AIS: Fifth year |  | >65 years | 42 | 5-Years post-stroke | 3,393 | Annual and 5-year cumulative costs according to 3-month mRS score | Mean | USD (2016) | 18,329.00 | (NR) |
|  | Patients with AIS |  | >65 years | 42 | 5-Years post-stroke | 3,393 | Annual and 5-year cumulative costs according to 3-month mRS score | Mean | USD (2016) | 117,576.00 | (NR) |
| Kim, 2019(30) | Patients with AIS | KNHANES database of the Korea Centers for Disease Control and Prevention (KCDC), and the NHIS-NSC database, and the Cause of Death Statistics of Statistics Korea | NR | NR | 2015 | 1,025,340 | Total direct costs of stroke in 2015 | Total | KRW (2015) | 1,118 billion | (NR) |
|  | Patients with AIS |  | NR | NR | 2014 | 1,025,340 | Total direct costs of stroke in 2014 | Total | KRW (2015) | 1,032 billion | (NR) |
|  | Patients with AIS |  | NR | NR | 2013 | 1,025,340 | Total direct costs of stroke in 2013 | Total | KRW (2015) | 964 billion | (NR) |
|  | Patients with AIS |  | NR | NR | 2012 | 1,025,340 | Total direct costs of stroke in 2012 | Total | KRW (2015) | 919 billion | (NR) |
|  | Patients with AIS |  | NR | NR | 2011 | 1,025,340 | Total direct costs of stroke in 2011 | Total | KRW (2015) | 874 billion | (NR) |
| **Denmark** | | | | | | | | | | | |
| Jakobsen*,* 2016(31) | Patients with AIS + AF | Danish National Patient Registry, the National Health Insurance Service Registry, Danish Registry of Medical Products Statistics, Civil Registration System, and Statistics Denmark | 78 | 54 | 3 Years Post-stroke | 21,673 | Total costs | Mean | USD (2012) | 20,507.00 | (NR) |
|  | Patients with AIS + AF |  | 78 | 54 | 3 Years Post-stroke | 21,673 | Inpatient hospitalization costs | Mean | USD (2012) | 19,066.00 | (NR) |
|  | Patients with AIS + AF |  | 78 | 54 | 3 Years Post-stroke | 21,673 | Outpatient hospital care costs | Mean | USD (2012) | 412.00 | (NR) |
|  | Patients with AIS + AF |  | 78 | 54 | 3 Years Post-stroke | 21,673 | Private practicing health professionals | Mean | USD (2012) | 668.00 | (NR) |
|  | Patients with AIS + AF |  | 78 | 54 | 3 Years Post-stroke | 21,673 | Prescribed medicine costs | Mean | USD (2012) | 361.00 | (NR) |
|  | Patients with AIS + AF |  | 78 | 54 | 3 Years Post-stroke | 21,673 | Total social care services | Mean | USD (2012) | 8,080.00 | (NR) |
|  | Patients with AIS + AF |  | 78 | 54 | 3 Years Post-stroke | 21,673 | Home help costs | Mean | USD (2012) | 3,952.00 | (NR) |
|  | Patients with AIS + AF |  | 78 | 54 | 3 Years Post-stroke | 21,673 | Nursing home costs | Mean | USD (2012) | 4,127.00 | (NR) |
|  | Patients with AIS + AF |  | 78 | 54 | 3 Years Post-stroke | 21,673 | Total costs | Mean | USD (2012) | 30,925.00 | (NR) |
|  | Patients with AIS + AF |  | 78 | 54 | Year 0 (incidence year) | 21,673 | Total costs | Mean | USD (2012) | 17,796.00 | (NR) |
|  | Patients with AIS + AF |  | 78 | 54 | Year 0 (incidence year) | 21,673 | Inpatient hospitalization costs | Mean | USD (2012) | 17,100.00 | (NR) |
|  | Patients with AIS + AF |  | 78 | 54 | Year 0 (incidence year) | 21,673 | Outpatient hospital care costs | Mean | USD (2012) | 532.00 | (NR) |
|  | Patients with AIS + AF |  | 78 | 54 | Year 0 (incidence year) | 21,673 | Private practicing health professionals | Mean | USD (2012) | 124.00 | (NR) |
|  | Patients with AIS + AF |  | 78 | 54 | Year 0 (incidence year) | 21,673 | Prescribed medicine costs | Mean | USD (2012) | 40.00 | (NR) |
|  | Patients with AIS + AF |  | 78 | 54 | Year 0 (incidence year) | 21,673 | Total social care services | Mean | USD (2012) | 1,905.00 | (NR) |
|  | Patients with AIS + AF |  | 78 | 54 | Year 0 (incidence year) | 21,673 | Home help costs | Mean | USD (2012) | 699.00 | (NR) |
|  | Patients with AIS + AF |  | 78 | 54 | Year 0 (incidence year) | 21,673 | Nursing home costs | Mean | USD (2012) | 1,206.00 | (NR) |
|  | Patients with AIS + AF |  | 78 | 54 | Year 0 (incidence year) | 21,673 | Total healthcare costs | Mean | USD (2012) | 19,989.00 | (NR) |
|  | Patients with AIS + AF |  | 78 | 54 | Year 1 after stroke | 21,673 | Total healthcare costs | Mean | USD (2012) | 3,354.00 | (NR) |
|  | Patients with AIS + AF |  | 78 | 54 | Year 1 after stroke | 21,673 | Hospitalization costs | Mean | USD (2012) | 2,826.00 | (NR) |
|  | Patients with AIS + AF |  | 78 | 54 | Year 1 after stroke | 21,673 | Outpatient hospital care costs | Mean | USD (2012) | 63.00 | (NR) |
|  | Patients with AIS + AF |  | 78 | 54 | Year 1 after stroke | 21,673 | Private practicing health professionals | Mean | USD (2012) | 297.00 | (NR) |
|  | Patients with AIS + AF |  | 78 | 54 | Year 1 after stroke | 21,673 | Prescribed medicine costs | Mean | USD (2012) | 168.00 | (NR) |
|  | Patients with AIS + AF |  | 78 | 54 | Year 1 after stroke | 21,673 | Total social care services | Mean | USD (2012) | 3,485.00 | (NR) |
|  | Patients with AIS + AF |  | 78 | 54 | Year 1 after stroke | 21,673 | Home help costs | Mean | USD (2012) | 1,704.00 | (NR) |
|  | Patients with AIS + AF |  | 78 | 54 | Year 1 after stroke | 21,673 | Nursing home costs | Mean | USD (2012) | 1,781.00 | (NR) |
|  | Patients with AIS + AF |  | 78 | 54 | Year 1 after stroke | 21,673 | Total costs | Mean | USD (2012) | 7,683.00 | (NR) |
|  | Patients with AIS + AF |  | 78 | 54 | Year 2 after stroke | 21,673 | Total costs | Mean | USD (2012) | 331.00 | (NR) |
|  | Patients with AIS + AF |  | 78 | 54 | Year 2 after stroke | 21,673 | Inpatient hospitalization costs | Mean | USD (2012) | 12.00 | (NR) |
|  | Patients with AIS + AF |  | 78 | 54 | Year 2 after stroke | 21,673 | Outpatient hospital care costs | Mean | USD (2012) | -177.00 | (NR) |
|  | Patients with AIS + AF |  | 78 | 54 | Year 2 after stroke | 21,673 | Private practicing health professionals | Mean | USD (2012) | 308.00 | (NR) |
|  | Patients with AIS + AF |  | 78 | 54 | Year 2 after stroke | 21,673 | Prescribed medicine costs | Mean | USD (2012) | 187.00 | (NR) |
|  | Patients with AIS + AF |  | 78 | 54 | Year 2 after stroke | 21,673 | Total social care services | Mean | USD (2012) | 3,403.00 | (NR) |
|  | Patients with AIS + AF |  | 78 | 54 | Year 2 after stroke | 21,673 | Home help costs | Mean | USD (2012) | 1,917.00 | (NR) |
|  | Patients with AIS + AF |  | 78 | 54 | Year 2 after stroke | 21,673 | Nursing home costs | Mean | USD (2012) | 1,486.00 | (NR) |
|  | Patients with AIS + AF |  | 78 | 54 | Year 2 after stroke | 21,673 | Total costs | Mean | USD (2012) | 5,176.00 | (NR) |
|  | Patients with AIS + AF |  | 78 | 54 | 3 Years Post-stroke | 21,673 | Productivity loss | Mean | USD (2012) | 2,338.0 | (NR) |
|  | Patients with AIS + AF |  | 78 | 54 | Year 0 (incidence year) | 21,673 | Productivity loss | Mean | USD (2012) | 288.0 | (NR) |
|  | Patients with AIS + AF |  | 78 | 54 | Year 1 after stroke | 21,673 | Productivity loss | Mean | USD (2012) | 843.0 | (NR) |
|  | Patients with AIS + AF |  | 78 | 54 | Year 2 after stroke | 21,673 | Productivity loss | Mean | USD (2012) | 1,442.0 | (NR) |
| Jennum*,* 2015(32) | Patients with AIS; before diagnosis | Danish National Patient Registry | NR | NR | 11 Years Post-stroke | 547,465 | Direct inpatient costs | Mean | EUR (NR) | 2,778.00 | (NR) |
|  | Control; before diagnosis |  | NR | NR | 11 Years Post-stroke | 2,129,024 | Direct inpatient costs | Mean | EUR (NR) | 2,018.00 | (NR) |
|  | Patients with AIS; before diagnosis |  | NR | NR | 11 Years Post-stroke | 398,140 | Direct inpatient costs | Mean | EUR (NR) | 8,431.00 | (NR) |
|  | Control; before diagnosis |  | NR | NR | 11 Years Post-stroke | 1,869,080 | Direct inpatient costs | Mean | EUR (NR) | 3,324.00 | (NR) |
|  | Patients with AIS; before diagnosis |  | NR | NR | 11 Years Post-stroke | 547,465 | Total healthcare costs | Mean | EUR (NR) | 5,248.00 | (NR) |
|  | Control; before diagnosis |  | NR | NR | 11 Years Post-stroke | 2,129,024 | Total healthcare costs | Mean | EUR (NR) | 2,018.00 | (NR) |
|  | Patients with AIS; before diagnosis |  | NR | NR | 11 Years Post-stroke | 398,140 | Total healthcare costs | Mean | EUR (NR) | 11,529.00 | (NR) |
|  | Control; before diagnosis |  | NR | NR | 11 Years Post-stroke | 1,869,080 | Total healthcare costs | Mean | EUR (NR) | 3,324.00 | (NR) |
|  | Patients with AIS; before diagnosis |  | NR | NR | 11 Years Post-stroke | 547,465 | Total healthcare costs | Mean | EUR (NR) | 3,231.00 | (NR) |
|  | Patients with AIS; before diagnosis |  | NR | NR | 11 Years Post-stroke | 398,140 | Total healthcare costs | Mean | EUR (NR) | 8,205.00 | (NR) |
|  | Patients with AIS; before diagnosis |  | NR | NR | (NR) | 547,465 | Foregone earnings | Mean | EUR (NR) | 2,470 | (NR) |
| **Finland** | | | | | | | | | | | |
| Raj, 2018(33) | Patients with TBI, ICH, SAH or AIS | Finnish Intensive Care Consortium database | 68 | 39 | 2003–2013 | 1,123 | University hospital costs | Mean | EUR (2013) | 15,819.00 | < 0.001 |
|  | Patients with TBI, ICH, SAH or AIS |  | 68 | 39 | 2003–2013 | 1,123 | Rehabilitation hospital | Mean | EUR (2013) | 16,579.00 | < 0.001 |
|  | Patients with TBI, ICH, SAH or AIS |  | 68 | 39 | 2003–2013 | 1,123 | Social security costs | Mean | EUR (2013) | 6,824.00 | < 0.001 |
|  | Patients with TBI, ICH, SAH or AIS |  | 68 | 39 | 2003–2013 | 1,123 | Total Costs | Mean | EUR (2013) | 39,222.00 | < 0.001 |
| **Canada** | | | | | | | | | | | |
| Tawfik, 2016(34) | Moderate to Severe IS Short Term | Institute for Clinical and Evaluative Sciences | NR | NR | NR | 1,150 | Total costs | Mean | CAD (2011) | 19,073.00 | (NR) |
|  | Moderate to Severe IS  Long Term |  | NR | NR | NR | 1,050 | Total costs | Mean | CAD (2011) | 87,724.00 | (NR) |
|  | Moderate to Severe IS  Short Term |  | NR | NR | NR | 1,150 | Total costs | Median | CAD (2011) | 16,946.00 | (NR) |
|  | Moderate to Severe IS  Long Term |  | NR | NR | NR | 1,050 | Total costs | Median | CAD (2011) | 53,138.00 | (NR) |
|  | Moderate to Severe IS  Short Term |  | NR | NR | 30-Days post-stroke | 1,150 | 30 day costs | Mean | CAD (2011) | 19,937.00 | (NR) |
|  | Moderate to Severe IS  Long Term |  | NR | NR | 30-Days post-stroke | 1,050 | 30 day costs | Mean | CAD (2011) | 4,525.00 | (NR) |
| **Russia** | | | | | | | | | | | |
| Kontsevaya*,* 2017(35) | Patients with AIS + T2DM | Federal statistics, Russian T2DM Epi studies, normative documents, meta-analyses, and local observational studies | NR | NR | 2014 | (NR) | Total economic burden (T2DM and AIS) | Mean | RUB (2014) | 7.0 billion | (NR) |
| **Mexico** | | | | | | | | | | | |
| Gongora-Rivera, 2021(36) | Patient with AIS | Mexican Endovascular Reperfusion Registry study | NR | NR | April 2020 | 21 Hospitals | Costs of EVT in public and private hospitals | Median | USD (NR) | 20,000.00 (Per EVT) | (NR) |
| **Taiwan** | | | | | | | | | | | |
| Lin, 2021(37) | First Year Patients with recurrent AIS | Taiwan’s National Health Insurance Research Database | NR | NR | 2 Years Post-stroke | 5,292 | Inpatient acute care costs | Median | USD (2011–2015) | 1,092.00 | (NR) |
|  | Second Year Patients with recurrent AIS |  | NR | NR | 2 Years Post-stroke | 4,163 | Inpatient acute care costs | Median | USD (2011–2015) | 1,151.00 | (NR) |
| **France** | | | | | | | | | | | |
| Cotté*,* 2016(38) | Patients with AF hospitalized for ischemic stroke | Programme de medicalization des systems d'information | 78 (11.4) | 47.1 | 2012 | 533,044 | Inpatient costs | Mean | EUR (NR) | 11,234.00 | (NR) |
| **Sweden** | | | | | | | | | | | |
| Banefelt*,* 2016(39) | Patients with hyperlipidaemia | Three national compulsory health registers governed by the National Board of Health and Welfare, and the Swedish Social Insurance Register | 56.5 | 27.25 | January  2006 – December 2012 | 688 | Indirect total costs by index cardiovascular (CV) event | Mean | EUR (2016) | 6,784.00 | <0.001 |

## AF: atrial fibrillation; AIS: acute ischemic stroke; ASU: Acute stroke unit; CAD: Canadian Dollar; CAD: Coronary artery disease; 95% CI: 95% confidence interval; CPI: consumer price index; EUR: Euro; EVT: endovascular thrombectomy; HF: heart failure; ICH: intracerebral hemorrhage; ICU: intensive care unit; IHS: in-hospital stroke; IQR: interquartile range; IV: intravenous; KRW: Korean Won; LOS: length of stay; LVO: large vessel occlusion; MI: myocardial infarction; MT: mechanical thrombectomy; PSD: post-stroke depression; RMB/CNY: Chinese Yuan; RUB: Ruble; SAH: subarachnoid hemorrhage; SD: standard deviation; SVO: Small-vessel occlusion; T2DM: type 2 diabetes mellitus; TBI: traumatic brain injury; tPA: tissue-type plasminogen activator; US: United States; USD: United States Dollar.

1. Kwok CS, Bains NK, Ford DE, Gomez CR, Hanley DF, Hassan AE, et al. Intra-arterial thrombolysis as adjunct to mechanical thrombectomy in acute ischemic stroke patients in the United States: A case control analysis. Journal of Stroke and Cerebrovascular Diseases. 2023;32(7) (no pagination).

2. Qureshi AI, Suri MFK, Huang W, Akinci Y, Chaudhry MR, Pond DS, et al. Annual Direct Cost of Dysphagia Associated with Acute Ischemic Stroke in the United States. Journal of Stroke and Cerebrovascular Diseases. 2022;31(5) (no pagination).

3. Aggarwal G, Patlolla SH, Aggarwal S, Cheungpasitporn W, Doshi R, Sundaragiri PR, et al. Temporal Trends, Predictors, and Outcomes of Acute Ischemic Stroke in Acute Myocardial Infarction in the United States. Journal of the American Heart Association. 2021:e017693.

4. Grandhi R, Ravindra VM, Ney JP, Zaidat O, Taussky P, de Havenon A. Investigating the "Weekend Effect" on Outcomes of Patients Undergoing Endovascular Mechanical Thrombectomy for Ischemic Stroke. J Stroke Cerebrovasc Dis. 2021;30(10):106013.

5. Khan SU, Khan MZ, Khan MU, Khan MS, Mamas MA, Rashid M, et al. Clinical and Economic Burden of Stroke Among Young, Midlife, and Older Adults in the United States, 2002-2017. Mayo Clinic Proceedings: Innovations, Quality and Outcomes. 2021;5(2):431-41.

6. Alqahtani F, Osman M, Harris AH, Hohmann SF, Alkhouli M. Mortality and functional outcomes of endovascular stroke therapy in the United States. Catheterization and Cardiovascular Interventions. 2021;97(3):470-4.

7. Wu C, Qin Y, Lin Z, Yi X, Wei X, Ruan Y, et al. Prevalence and Impact of Aphasia among Patients Admitted with Acute Ischemic Stroke. J Stroke Cerebrovasc Dis. 2020;29(5):104764.

8. Yousufuddin M, Moriarty JP, Lackore KA, Zhu Y, Peters JL, Doyle T, et al. Initial and subsequent 3-year cost after hospitalization for first acute ischemic stroke and intracerebral hemorrhage. Journal of the Neurological Sciences. 2020;419:117181.

9. Alkhouli M, Alqahtani F, Aljohani S, Alvi M, Holmes DR. Burden of Atrial Fibrillation-Associated Ischemic Stroke in the United States. JACC Clin Electrophysiol. 2018;4(5):618-25.

10. Dave A, Cagniart K, Holtkamp MD. A Case for Telestroke in Military Medicine: A Retrospective Analysis of Stroke Cost and Outcomes in U.S. Military Health-Care System. Journal of Stroke and Cerebrovascular Diseases. 2018;27(8):2277-84.

11. Mu F, Hurley D, Betts KA, Messali AJ, Paschoalin M, Kelley C, et al. Real-world costs of ischemic stroke by discharge status. Current Medical Research and Opinion. 2017;33(2):371-8.

12. Mukherjee K, Kamal KM. Impact of atrial fibrillation on inpatient cost for ischemic stroke in the USA. International Journal of Stroke. 2019;14(2):159-66.

13. Chaudhry SA, Afzal MR, Chaudhry BZ, Zafar TT, Safdar A, Kassab MY, et al. Rates of Adverse Events and Outcomes among Stroke Patients Admitted to Primary Stroke Centers. Journal of Stroke and Cerebrovascular Diseases. 2016;25(8):1960-5.

14. Johnson BH, Bonafede MM, Watson C. Short- and longer-term health-care resource utilization and costs associated with acute ischemic stroke. ClinicoEconomics and Outcomes Research. 2016;8:53-61.

15. Kumar N, Khera R, Pandey A, Garg N. Racial Differences in Outcomes after Acute Ischemic Stroke Hospitalization in the United States. Journal of Stroke and Cerebrovascular Diseases. 2016;25(8):1970-7.

16. Sonig A, Lin N, Krishna C, Natarajan SK, Mokin M, Hopkins LN, et al. Impact of transfer status on hospitalization cost and discharge disposition for acute ischemic stroke across the US. Journal of Neurosurgery. 2016;124(5):1228-37.

17. Tong X, George MG, Gillespie C, Merritt R. Trends in hospitalizations and cost associated with stroke by age, United States 2003-2012. International Journal of Stroke. 2016;11(8):874-81.

18. Agarwal S, Menon V, Jaber WA. Outcomes After Acute Ischemic Stroke in the United States: Does Residential ZIP Code Matter? Journal of the American Heart Association. 2015;4(3) (no pagination).

19. Pan X, Simon TA, Hamilton M, Kuznik A. Comparison of costs and discharge outcomes for patients hospitalized for ischemic or hemorrhagic stroke with or without atrial fibrillation in the United States. J Thromb Thrombolysis. 2015;39(4):508-15.

20. Su M, Pan D, Zhao Y, Chen C, Wang X, Lu W, et al. The direct and indirect effects of length of hospital stay on the costs of inpatients with stroke in Ningxia, China, between 2015 and 2020: A retrospective study using quantile regression and structural equation models. Frontiers in public health. 2022;10:881273.

21. Doan J, Ko F. Anesthetic Choices and Postoperative Delirium Incidence: Propofol vs Sevoflurane. Journal of Clinical Outcomes Management. 2022;29(6):199-205.

22. Lu Y, Sun W, Shen Z, Sun W, Liu R, Li F, et al. Regional Differences in Hospital Costs of Acute Ischemic Stroke in China: Analysis of Data From the Chinese Acute Ischemic Stroke Treatment Outcome Registry. Frontiers in Public Health. 2021;9:783242.

23. Xin J, Huang X, Liu C, Huang Y. Coronavirus disease 2019 is threatening stroke care systems: a real-world study. BMC health services research. 2021;21(1):288.

24. Liu H, Zhu C, Cao J, Jiao J, Song B, Jin J, et al. Hospitalization costs among immobile patients with hemorrhagic or ischemic stroke in China: a multicenter cross-sectional study. BMC health services research. 2020;20(1):905.

25. Yang Y, Man X, Nicholas S, Li S, Bai Q, Huang L, et al. Utilisation of health services among urban patients who had an ischaemic stroke with different health insurance-a cross-sectional study in China. BMJ Open. 2020;10(10) (no pagination).

26. Kong D, Liu X, Lian H, Zhao X, Zhao Y, Xu Q, et al. Analysis of Hospital Charges of Inpatients with Acute Ischemic Stroke in Beijing, China, 2012-2015. Neuroepidemiology. 2018;50(1-2):63-73.

27. Wen L, Wu J, Feng L, Yang L, Qian F. Comparing the economic burden of ischemic stroke patients with and without atrial fibrillation: a retrospective study in Beijing, China. Current Medical Research and Opinion. 2017;33(10):1789-94.

28. Li J, Luo W. Hospitalization expenses of acute ischemic stroke patients with atrial fibrillation relative to those with normal sinus rhythm. Journal of Medical Economics. 2017;20(2):114-20.

29. Kim SE, Lee H, Kim JY, Lee KJ, Kang J, Kim BJ, et al. Three-month modified Rankin Scale as a determinant of 5-year cumulative costs after ischemic stroke: An analysis of 11,136 patients in Korea. Neurology. 2020;06.

30. Kim JYK, K. Kang, J. Koo, J. Kim, D. H. Kim, B. J. Kim, W. J. Kim, E. G. Kim, J. G. Kim, J. M. Kim, J. T. Kim, C. Nah, H. W. Park, K. Y. Park, M. S. Park, J. M. Park, J. H. Park, T. H. Park, H. K. Seo, W. K. Seo, J. H. Song, T. J. Ahn, S. H. Oh, M. S. Oh, H. G. Yu, S. Lee, K. J. Lee, K. B. Lee, K. Lee, S. H. Lee, S. J. Jang, M. U. Chung, J. W. Cho, Y. J. Choi, K. H. Choi, J. C. Hong, K. S. Hwang, Y. H. Kim, S. E. Lee, J. S. Choi, J. Kim, M. S. Kim, Y. J. Seok, J. Jang, S. Han, S. Han, H. W. Hong, J. H. Yun, H. Lee, J. Bae, H. J. Executive summary of stroke statistics in Korea 2018: A report from the epidemiology research council of the korean stroke society. Journal of Stroke. 2019;21(1):42-59.

31. Jakobsen M, Kolodziejczyk C, Fredslund EK, Poulsen PB, Dybro L, Johnsen SP. Societal Costs of First-Incident Ischemic Stroke in Patients with Atrial Fibrillation-A Danish Nationwide Registry Study. Value in Health. 2016;19(4):413-8.

32. Jennum P, Iversen HK, Ibsen R, Kjellberg J. Cost of stroke: a controlled national study evaluating societal effects on patients and their partners. BMC health services research. 2015;15:466.

33. Raj RB, S. Reinikainen, M. Hoppu, S. Laitio, R. Ala-Kokko, T. Curtze, S. Skrifvars, M. B. Costs, outcome and cost-effectiveness of neurocritical care: A multi-center observational study. Critical Care. 2018;22(1) (no pagination).

34. Tawfik A, Wodchis WP, Pechlivanoglou P, Hoch J, Husereau D, Krahn M. Using Phase-Based Costing of Real-World Data to Inform Decision-Analytic Models for Atrial Fibrillation. Applied Health Economics and Health Policy. 2016;14(3):313-22.

35. Kontsevaya AB, Y. Economic burden of type 2 diabetes and its cardiovascular complications in Russian Federation. European Heart Journal. 2017;38(Supplement 1):712.

36. Gongora-Rivera FG-A, A. Marquez-Romero, J. M. Identification of Barriers to Access Endovascular Treatment for Acute Ischemic Stroke in the Health Care System of Mexico: Results From a National Survey Among Endovascular Neurologists. Frontiers in Neurology. 2021;12(no pagination).

37. Lin FJ, Jhang JG, Kuo YH, Yeh E, Pinto L, Wu CC. PCV17 Cost Analysis of Myocardial Infarction and Ischemic Stroke Using the National Claims Database in Taiwan. Value in Health. 2021;24(Supplement 1):S69.

38. Cotte FE, Chaize G, Gaudin AF, Samson A, Vainchtock A, Fauchier L. Burden of stroke and other cardiovascular complications in patients with atrial fibrillation hospitalized in France. Europace. 2016;18(4):501-7.

39. Banefelt J, Hallberg S, Fox KM, Mesterton J, Paoli CJ, Johansson G, et al. Work productivity loss and indirect costs associated with new cardiovascular events in high-risk patients with hyperlipidemia: estimates from population-based register data in Sweden. European Journal of Health Economics. 2016;17(9):1117-24.
